# Geometry-aware graph attention networks to explain single-cell chromatin states and gene expression with SEAGALL

**DOI:** 10.1186/s13059-026-04066-2

**Published:** 2026-04-23

**Authors:** Gabriele Malagoli, Patrick Hanel, Anna Danese, Guy Wolf, Maria Colomé-Tatché

**Affiliations:** 1 Institute of Computational Biology, Computational Health Center, Helmholtz Munich, Munich, Germany; 2 Biomedical Center, Division of Physiological Chemistry, Faculty of Medicine, Ludwig-Maximilians-Universität Munich, Munich, Germany; 3 Mila - Quebec Artificial Intelligence Institute, Montréal, Québec Canada; 4 Institute of Stem Cell Research, Helmholtz Munich, Munich, Germany; 5 Chair of Cell Biology and Anatomy, Biomedical Center (BMC), Faculty of Medicine, Ludwig-Maximilians-Universität Munich, Munich, Germany; 6 Department of Mathematics and Statistics, Université de Montréal, Montréal, Québec Canada; 7 Hospital del Mar Research Institute (HMRIB), Barcelona, Spain

## Abstract

High-throughput single-cell sequencing is widely used to study cell identity. We present SEAGALL (Single-cell Explainable Geometry-Aware Graph Attention Learning pipeLine), a deep learning method to quantify the impact of molecular features on cellular phenotype, based on geometry-regularised autoencoders (GRAE) and explainable graph attention networks (X-GAT). The GRAE embeds the data into a latent space to build a reliable cell-cell graph. The GAT is trained to learn the annotations and XAI is used to explain the predictions, unravelling the features driving cell identity. SEAGALL extracts specific and stable signatures from multiple omics experiments, going beyond differential marker genes.

**Supplementary Information:**

The online version contains supplementary material available at 10.1186/s13059-026-04066-2.

## Background

**Fig. 1:**
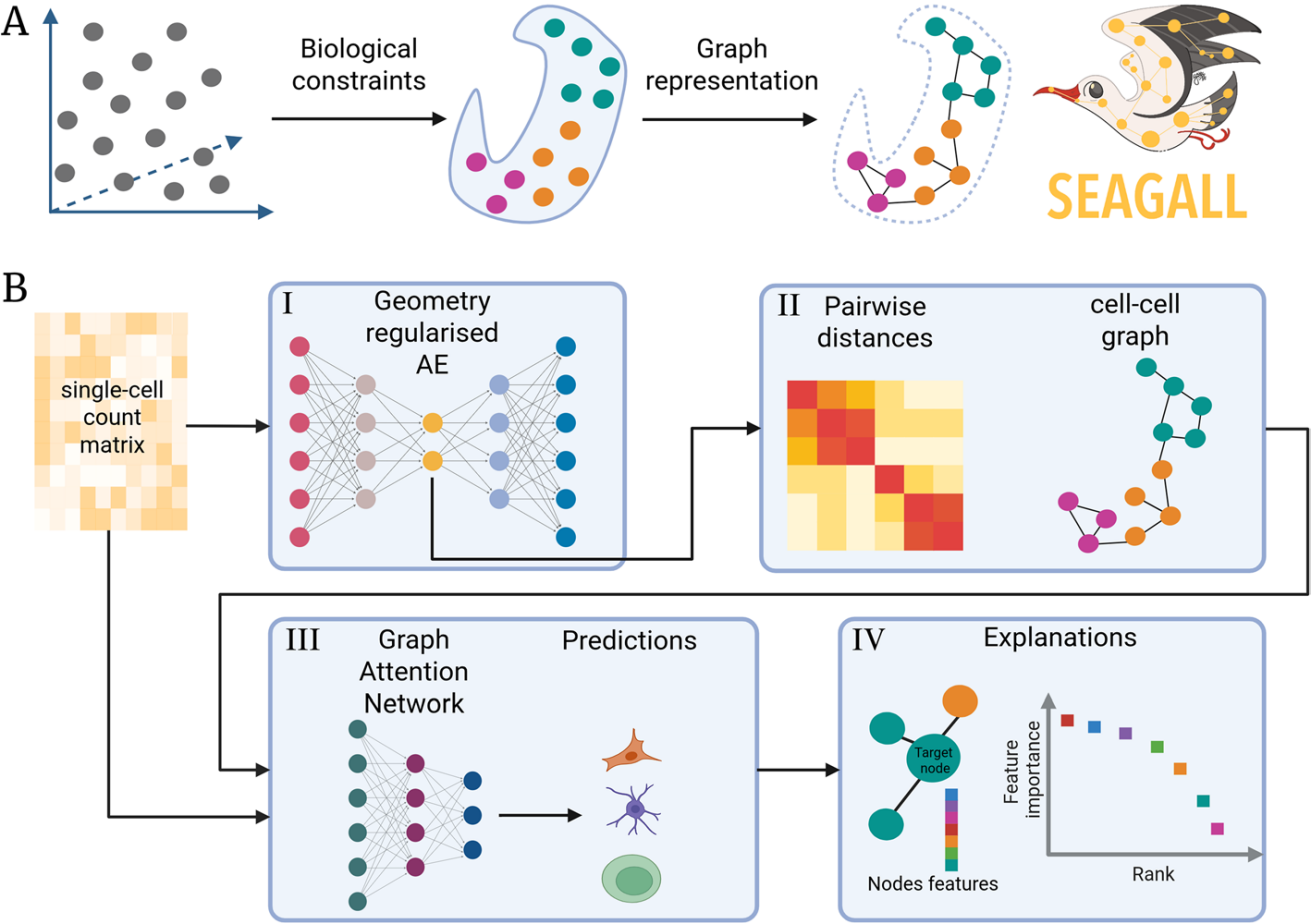
**A** Without any constraint, the measurement of molecular features can take any arbitrary value (left). In reality, the gene regulatory network imposes constraints that define the cell type and the possible values of the variables, thereby defining a manifold where the data live (centre). As a proxy for the manifold, a cell-cell graph (right) can be used. **B** The SEAGALL model. The initial count matrix is reduced with a GRAE to preserve data geometry, i.e. both local and global structure of the data (I). Within the latent space, we compute pairwise distances between cells to build the cell-cell graph (II). The graph and the count matrix are subsequently used as input to a GAT classifier, whose predictions (III) are then explained to identify the most relevant features for each cell type (IV). Figure created with BioRender

Single-cell sequencing technologies have provided a breakthrough in molecular biology by allowing the measurement of transcriptomic and epigenomic profiles at high read depth and single-cell resolution. Many reproducible and ready-to-use kits have become common and affordable, leading to a substantial increase in interest in this field. For instance, single-cell RNA sequencing (scRNA-seq) [1], also known as gene expression (GEX), or single-cell Assay for Transposase-Accessible Chromatin using sequencing (scATAC-seq) [2] can be performed using readily available kits commercialised by 10X Genomics, following their well-described protocols. A new step towards understanding molecular biology is the possibility of performing multi-omic single-cell sequencing, which allows the simultaneous measurement of multiple modalities within the same single-cell. Among others, the 10X Genomics Multiome Platform, which quantifies chromatin openness and the transcriptome of single nuclei, also allows such measurements. The standard analysis of single-cell data involves low-dimensional embedding, followed by cell clustering and cell-type identification [3]. The common assumption, known as the “manifold hypothesis” [4, 5], is that high-dimensional data lie on a latent, unknown manifold of lower dimension than the observed space. In single-cell biology, we measure tens of thousands of variables, such as genes (for gene expression measurements) or genomic loci (for epigenomic measurements). These features cannot take any possible value; instead, they vary within well-defined ranges constrained by biological mechanisms, such as gene regulatory networks [6]. These constraints define the underpinning manifold whose exact equations are unknown. A single-cell experiment can be seen as a method to sample (cells) from this manifold. From the distances between cells, we can create a graph that resembles the manifold as accurately as possible (Fig. 1A).

Many tools exist to perform single-cell analysis [3], yet they show several limitations. First of all, they are, in general, omic-specific [7–12], relying on omic-specific assumptions, and forcing the users to choose a different tool for each omic. Moreover, most standard tools compute distances in a low-dimensional linear space, such as Principal Components Analysis (PCA) or Independent Component Analysis (ICA) [7, 9, 10, 13–15]. These linear assumptions lead to the loss of the intrinsic nonlinearity present in biological data sets and prevent the discovery of complex insights between features and cells. Due to these shortcomings, autoencoders (AEs) [16] have recently become very popular for their ability to learn the input and embed it in a nonlinear fashion [11, 12, 16–18]. Indeed, their strongest characteristic is the ability to take into account nonlinear dependencies within the data sets without making strong data assumptions. Yet, autoencoders often fail to represent the intrinsic data structure [5], such as topology or geometry. To better fit single-cell data, omic-specific AE have been developed; they assume a probability distribution from which the data are sampled and use variational AE to embed the data sets [11, 12, 17, 18]. As a consequence, a specific AE is needed for each modality, whose number continues to increase. Finally, another important aspect of single-cell data analysis is the identification of features defining cell identity. The standard method for investigating important features, such as genes or peaks, in each cell group is differential analysis (DA). It consists of computing the distributions of the features in the different treatments, conditions or cell types to then quantify the difference between these distributions, giving as a result a list of features ranked by the most different to the least ones. This approach will output features which are different between two groups of cells, but there is no guarantee that they are also relevant and important within each group.

To address these limitations, we developed SEAGALL (Single-cell ExplAinable Geometry-Aware Graph Attention Learning pipeLine), a deep learning method based on manifold learning and explainable AI for downstream analysis of single-cell data sets. SEAGALL first learns a low-dimensional embedding of the cells using a graph-regularised autoencoder (GRAE) [5] (Fig. 1B I). This embedding preserves both the local and global structure of the data without making any assumptions about the data-generating process. Then the tool computes the cell-cell k-nearest neighbours (k-NN) graph on that low-dimensional space (Fig. 1B II), which is used as input to a graph attention network (GAT) [19, 20] together with the count matrix defining feature vectors of the nodes. The GAT classifies the cells into predefined cell types or states (Fig. 1B III), and the final output of SEAGALL is the explanations of the model, i.e. the set of input features which are the most important for the prediction of the labels [21] (Fig. 1B IV). We applied our new method to ten different single-cell data sets spanning three omics (scRNA-seq, scATAC-seq, scChIP-seq [22]) showing that it is able to reconstruct and embed the data, explain the cell types beyond common marker genes and extracting stable and specific features that are not identified by standard differential analysis, usually performed with Scanpy [7]/Seurat [23] or SnapATAC2 [24]/Signac [9] depending on feature spaces.

## Results

### The SEAGALL model

We can represent a single-cell experiment with a $N \times F$ count matrix, i.e *N* cells in an *F*-dimensional space, commonly called point cloud. Without constraints, the point would span a homogeneous volume in space. Yet constraints do exist, imposed for example by gene regulatory networks; therefore, the data do not occupy a homogeneous volume, but rather live on a manifold [4] (Fig. 1A), whose equations are unknown. However, the manifold is high-dimensional, making it difficult to compute distances on it due to the curse of dimensionality.

Hence, the first step of SEAGALL is to learn a low-dimensional representation of the data that conserves the intrinsic geometry of the manifold, exploiting recent developments in geometry-regularised autoencoders (GRAE) [5] (Methods) (Fig. 1B I). The GRAE first applies a kernel method named PHATE [25] to learn the geometry of the data and uses it to regularise the structure of its latent space. Within the latent space of the GRAE, it is now possible to compute reliable pairwise distances between cells in order to create a cell-cell graph (Fig. 1B II). In the next step of SEAGALL, the cell-cell graph is used as input to a graph attention network [19, 20] (GAT) (Fig. 1B III), a graph neural network [26] (GNN) with an attention mechanism on the edges. The GAT is applied to learn cell labels based on previous annotations (e.g., cell type) or knowledge (e.g., tumour type, treatment, etc.). Therefore, the model does not apply any clustering approach to produce a new grouping of the cells, but instead learns the genomic features driving the labels. In this scenario, the classification of a cell depends on its neighbourhood, via the joint embedding of *k* feature vectors, if the cell has degree *k* (see Methods). The attention mechanism is important for dynamically learning the relevance of each edge: spurious edges are ignored, allowing the model to focus on the important ones. This approach is chosen for its ability to infer the underlying nonlinear dependencies that govern the relationship between molecular data and phenotype, and to quantify the impact of each feature on the label, such as cell type, disease state, or treatment. To evaluate the contribution of the geometry regularisation, we have performed an ablation study, systematically replacing it with linear and nonlinear methods. We have tested robustness and the ability of preserving the biological signal of the GRAE together with a topological autoencoder (TAE) [27], a standard autoencoder (AE), a variational one (VAE), PeakVI [12], scVI [11], a VAE tailored to interpret genomic data (siVAE) [28] and linear PCA (Methods). Then, we compared results obtained with a GAT and a graph convolutional neural network (GCN) (Methods). The ablation study was carried out using six count matrices, two from scRNA-seq and four from scATAC-seq (Additional file 1: Tables S1 and S2 for the cell type composition and dimensions).

### Geometrical regularised autoencoders best recover corrupted data

**Fig. 2:**
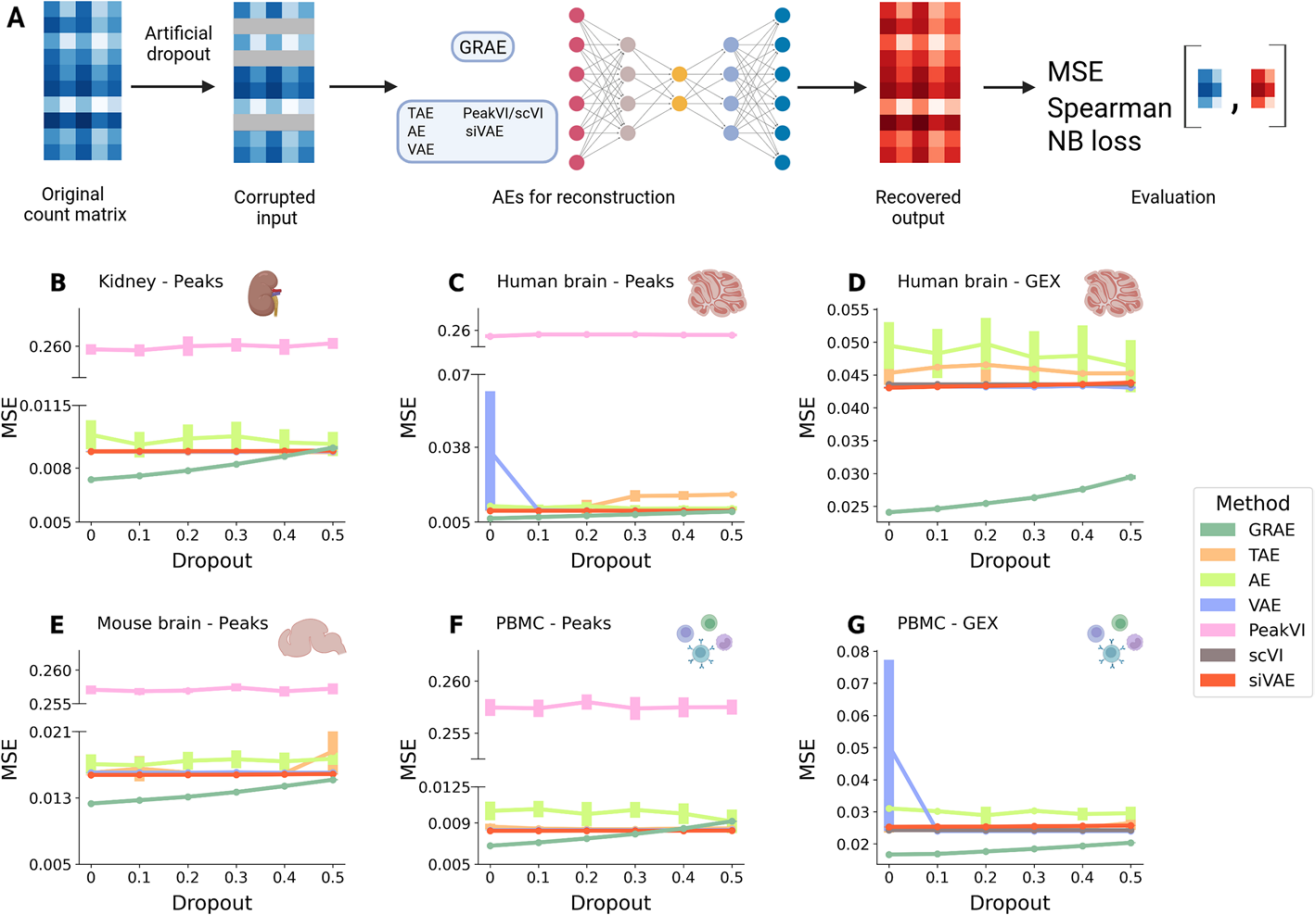
**A** Schematic of the ablation study to test the ability of the AEs to reconstruct input data: the data are corrupted with artificial dropout, and the AEs reconstruct them. We evaluated the performance by computing the MSE between the recovered output and the original count matrix. Graphic created with BioRender. **B**–**G** Average MSE at different levels of artificial dropout for each AE. Each point is the average of ten runs, and the height of the error bar represents three times the uncertainty on the mean

To measure the ability of the autoencoders to retrieve corrupted data, we applied a variable dropout to the six RNA and ATAC count matrices and trained each AE on the faulty data to then measure the mean squared error (MSE) between the original and the reconstructed data (Fig. 2A, Methods).

The GRAE outperforms all the methods, achieving the minimum MSE at every dropout level (Fig. 2B–G), except for the highest dropout on the peaks of the PBMC data [29]. In particular, the geometry-regularised AE is better than the two omic-specific AEs, scVI and PeakVI. Since MSE can be sensitive to outliers, we also measured the MSE considering only the most covered features [30], computing the coverage either on the input data or on the reconstructed matrix, the negative binomial loss [11], and the Spearman coefficient between the original and the reconstructed matrices (Methods). According to these metrics, the GRAE systematically outperforms all other AEs across all data sets and corruption levels (Additional file 2: Figs. S1–S3).

### Geometrical regularisation best matches the biological structure of the cell-cell graph

**Fig. 3:**
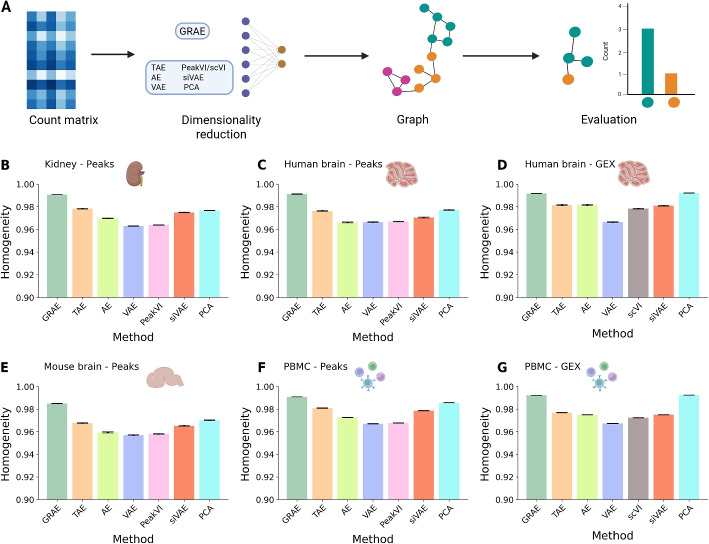
**A** Schematic of the ablation study to test the conservation of the biological signal: each count matrix is embedded using the different AEs or by PCA, the cell-cell graph is computed from the latent space, and its homogeneity is used to evaluate the performance of the AE. Graphic created with BioRender. **B**–**G** Homogeneity of the k-NN using the different embedding methods. The height of the bar represents the average homogeneity across runs, and the error bars represent three times the uncertainty on the mean

We also quantified the ability of each latent space to preserve biological structure by measuring the homogeneity of the k-NN graphs built from the AEs latent spaces or PCA (Fig. 3A, Methods). We assume that a good latent space yields a k-NN graph where neighbours of a node belong to the same cell type. The more homogeneous the neighbourhood, the more effectively the AE can link cells that share the same biological function. To quantify this, we trained each AE, computed k-NN (k=15) graphs from their latent spaces, and calculated a homogeneity metric (i.e., the proportion of cells sharing a type within a neighbourhood). None of the embedding methods can outperform the GRAE (Fig. 3B–G, Additional file 1: Table S3), independently of the dropout level (Additional file 2: Fig. S4).

In conclusion, the GRAE, which applies a geometrical regularisation to the loss function, outperforms all other methods in reconstructing the initial input, even with added noise in the data. It also performs either better or equal to the other methods at recovering the biological composition in the latent space across all considered noise levels.

### Geometry-aware graph attention networks achieve the best classification performance

**Fig. 4:**
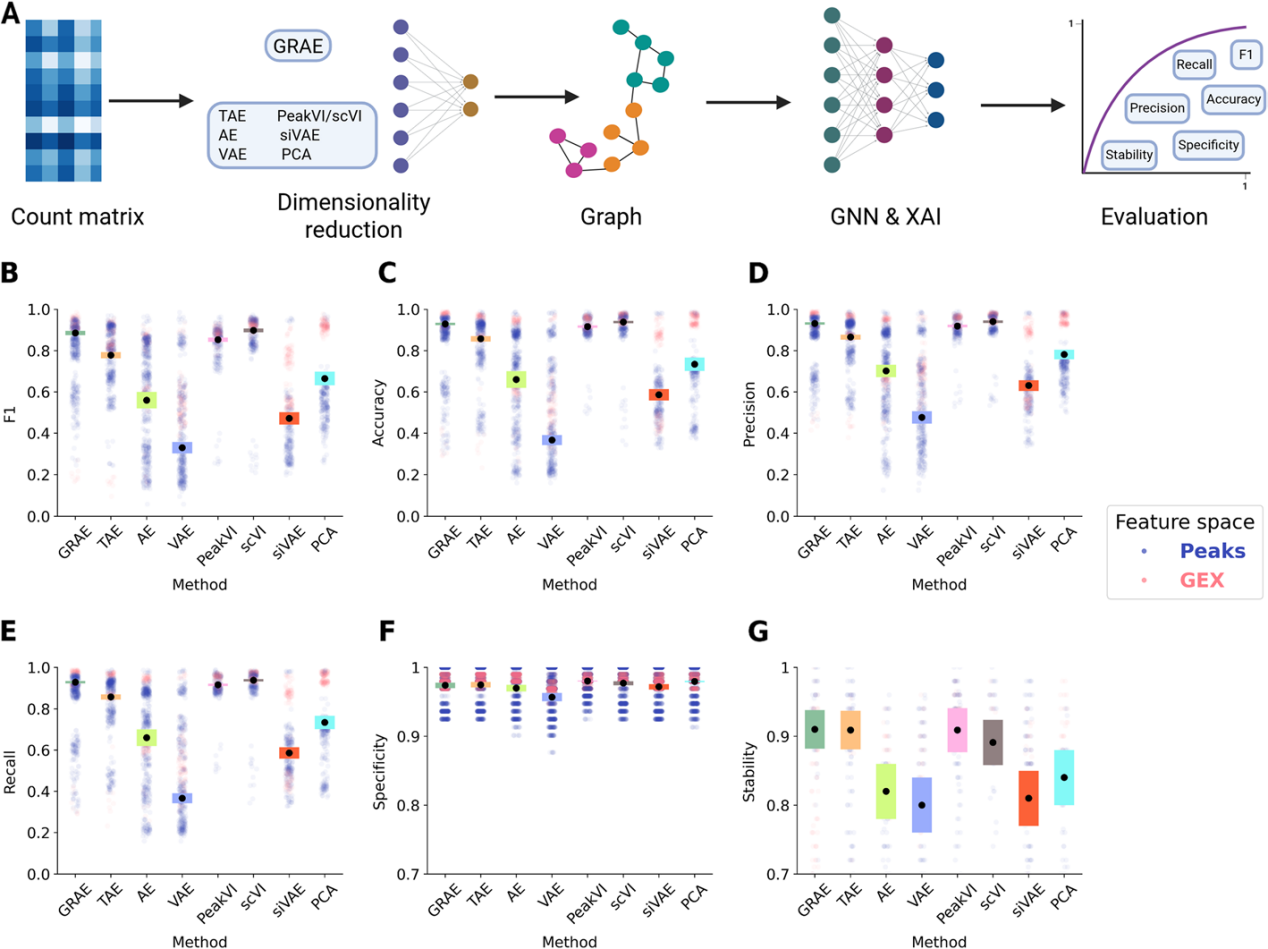
**A** Schematic of the ablation study to test the quality of each latent space for learning the cell annotation: After computing the k-NN graph, we trained a GNN classifier and applied the GNNExplainer; we then computed the shown metrics to evaluate the models. Graphic created with BioRender. **B**–**E** Classification performance of the GAT classifier varying the embedding methods. Each black dot represents the mean across 50 runs, and the height of the bars represents three times the standard deviation of the mean. **F**–**G** Specificity and stability of the explanations. The vertical axes start from 0.7 for visualisation purposes. In all panels, each pale plot represents the point contributing to the mean, coloured by data modality. PeakVI is run only on peak data sets, and scVI is run only on GEX data sets; all other methods are run on all data sets

Lastly, we tested the performance of the different embedding strategies combined with GNN classifiers, namely GAT [20] and GCN [31]. We applied four metrics to assess classification performance (accuracy, F1 score, precision and recall) and two metrics to measure the quality of the explanations (specificity and stability) (Fig. 4A). The specificity quantifies the uniqueness of the explanations, and stability quantifies how much they vary over different initialisations (Methods). The final goal of our model is to explain the assigned labels; to achieve this it is crucial that the model can learn them. Therefore, classification metrics are critical for testing whether the methods can understand the underlying biology. We did not observe any statistical differences in performance between the GAT and the GCN for F1, accuracy, precision, recall, specificity, and stability (Additional file 2: Fig. S5, Additional file 1: Table S4). We opted for the attention mechanism because it is theoretically more reliable [20, 32]. The graph derived from the GRAE latent space makes the GNNs achieve the best classification and explanations performance (Fig. 4B–G, Additional file 1: Table S5), in both the peaks (Additional file 1: Table S6) and GEX spaces (Additional file 1: Table S7), compared to the other tested embedding methods. We tested the final combination of GRAE and GAT on the scChIP-seq data set and showed very high performance for that data type, including accuracy, F1, precision, recall, as well as specificity and stability of the discovered features (Additional file 2: Fig. S6).

In conclusion, these results indicate that GRAE combined with a GAT classifier best learns cell biological annotations and derives the most stable and specific explanations.

### SEAGALL retrieves stable, specific and unbiased features

**Fig. 5:**
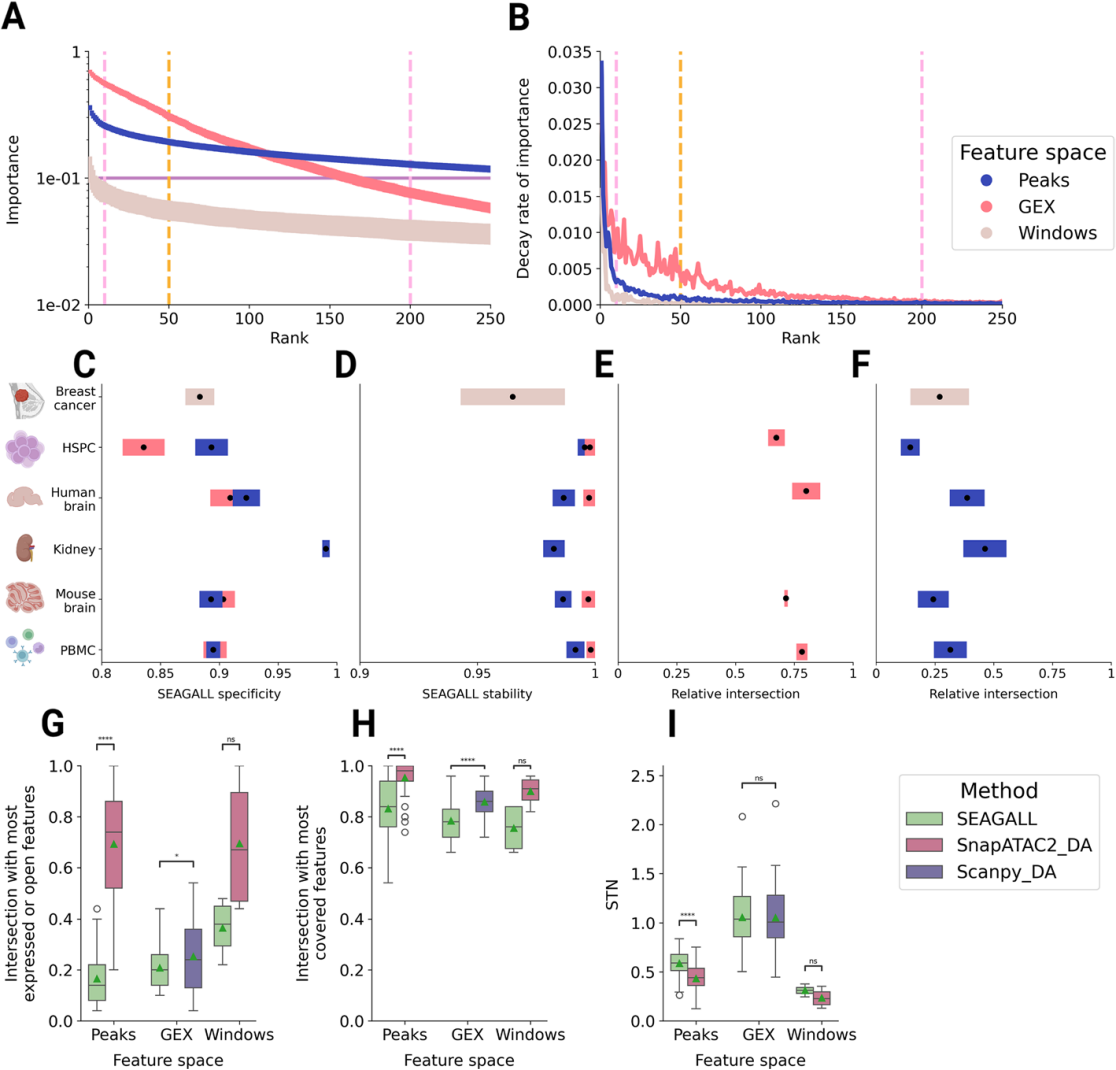
**A** Rank-importance distribution of the features according to the explainer. Average across data sets and cell type. Vertical dashed lines highlight the 10–200 feature interval where importance is stable. **B** Decay rate of the importance of the features, legend as in A. **C**, **D** Specificity (left), stability (right) of the features obtained with SEAGALL in the ten data sets. Icons created with BioRender. **E**, **F** Similarity between the SEAGALL features and the differential ones, computed with Scanpy for GEX and with SnapATAC2 for peaks and windows. Colour legend as in panel **B**. **G** Distribution of the overlap between the most expressed or open features and SEAGALL (green), SnapATAC2 (red) and Scanpy (violet). **H** Distribution of the overlap between the most covered features and SEAGALL (green), SnapATAC2 (red), and Scanpy (violet). **I** Distribution of the signal-to-noise (STN) ratio and SEAGALL (green), SnapATAC2 (red) and Scanpy (violet)

Given these results, the final SEAGALL model consists of the GRAE to embed the data and build the graph, and the GAT to classify cells (Fig. 5A). However, the final and crucial step is to explain the predictions. This point is critical, as it shifts the focus from predictive performance to model interpretability, making the tool more translational and useful for providing potentially novel biological insights. Once the GAT is trained on the geometry-aware graph, SEAGALL investigates which features drive the predictions of the model. To do this, it applies a mask-based graph neural network explainer, known as GNNExplainer [21]. This way, SEAGALL serves as an alternative or complementary method to differential analysis, explaining a cell phenotype using an ML approach by perturbing the inputs and quantifying their impact on the output. We compared the features obtained by differential analysis, performed with Scanpy [7] (for GEX data) or SnapATAC2 [24] (for ATAC and ChIP data), with those extracted by our tool. The explainer in SEAGALL identifies the subset of node features (and node links) that are most important for predicting the label of a node. The importance is defined as the mutual information between a feature and the predictions (Methods).

The distribution of feature importance drops rapidly with rank, especially in GEX data. For both genes and peaks, at around the two hundredth feature, the importance drops one order of magnitude (Fig. 5A, Additional file 2: Fig. S7A). Therefore, we suggest using fewer features for downstream analysis. For the scChIP-seq data set, windows show different behaviour: the maximum importance is quite smaller than in peaks and GEX, and the importance decay is slower (Fig. 5A, Additional file 2: Fig. S7A). We speculate that because of the noisier and sparser nature of the data, each individual feature has a lower impact on the final prediction. However, the decay rate of the importance, computed as the absolute value of the derivative of the importance by the rank, shows that for all three feature spaces the importance of the features does not change any more after rank two hundred (Fig. 5B, Additional file 2: Fig. S7B). Windows have a slower decay, again suggesting that each individual window has a lower impact on the results. We selected fifty features for the downstream analysis to quantify the impact of technical biases on the DA and XAI, as fifty is close to the typical number of proteins detectable in a CyTOF [33] experiment or the number of markers that can be used in a FACS [34] experiment. We measured the stability and specificity of the features obtained with SEAGALL (Methods). We found that the method can extract cell-type-specific (Fig. 5C) and highly stable (Fig. 5D) features across all the count matrices we tested, independent of the number of selected features (Additional file 2: Fig. S8A, B). This indicates that the model can consistently capture and explain the dataset structure to suggest key degrees of freedom for downstream analysis and wet-lab experiments, linking the deep learning method to real-world information. Notably, the features obtained with SEAGALL using XAI (XAIFs) consistently differ from the differential features (DAFs) obtained with Scanpy/SnapATAC2 (Fig. 5E, F, Additional file 2: Fig. S9, Fig. S8C, D), providing a potential for the discovery of novel data characteristics. This is due to the nonlinearity and awareness of the geometry of the model we propose. To test the effect of the inclusion of the geometry and attention in our model, we conducted an ablation study by replacing either the GRAE with an AE, or the GAT with a standard neural network (NN), and we showed that this leads to different explanations (Additional file 2: Fig. S10). A direct comparison between XAIFs and DAFs shows that the former are less biased by high openness or expression and coverage (Methods) (Fig. 5G, H). This is particularly strong with peaks. Nevertheless, the lower biases of XAIFs are not traded off with a higher noise: the signal-to-noise (STN) ratio of XAIFs is, on average, either the same or higher than that of DAFs (Fig. 5I).

### SEAGALL identifies chromatin priming states and known cell type predictors

To study the biological significance of our results, we examined features identified only by SEAGALL and not by differential analysis, namely, their expression or openness, which were not differentially expressed according to Scanpy (for GEX) or SnapATAC2 (for scATAC-seq and scChIP-seq). For the scATAC-seq feature spaces, to link genomic loci to transcription factors (TFs), we run motif analysis on the top-ranked features using HOMER [35]. HOMER takes as input a set of genomic intervals and identifies enriched motifs, i.e. recurrent patterns of bases, and it checks whether these patterns match known motifs of TF binding.

**Fig. 6:**
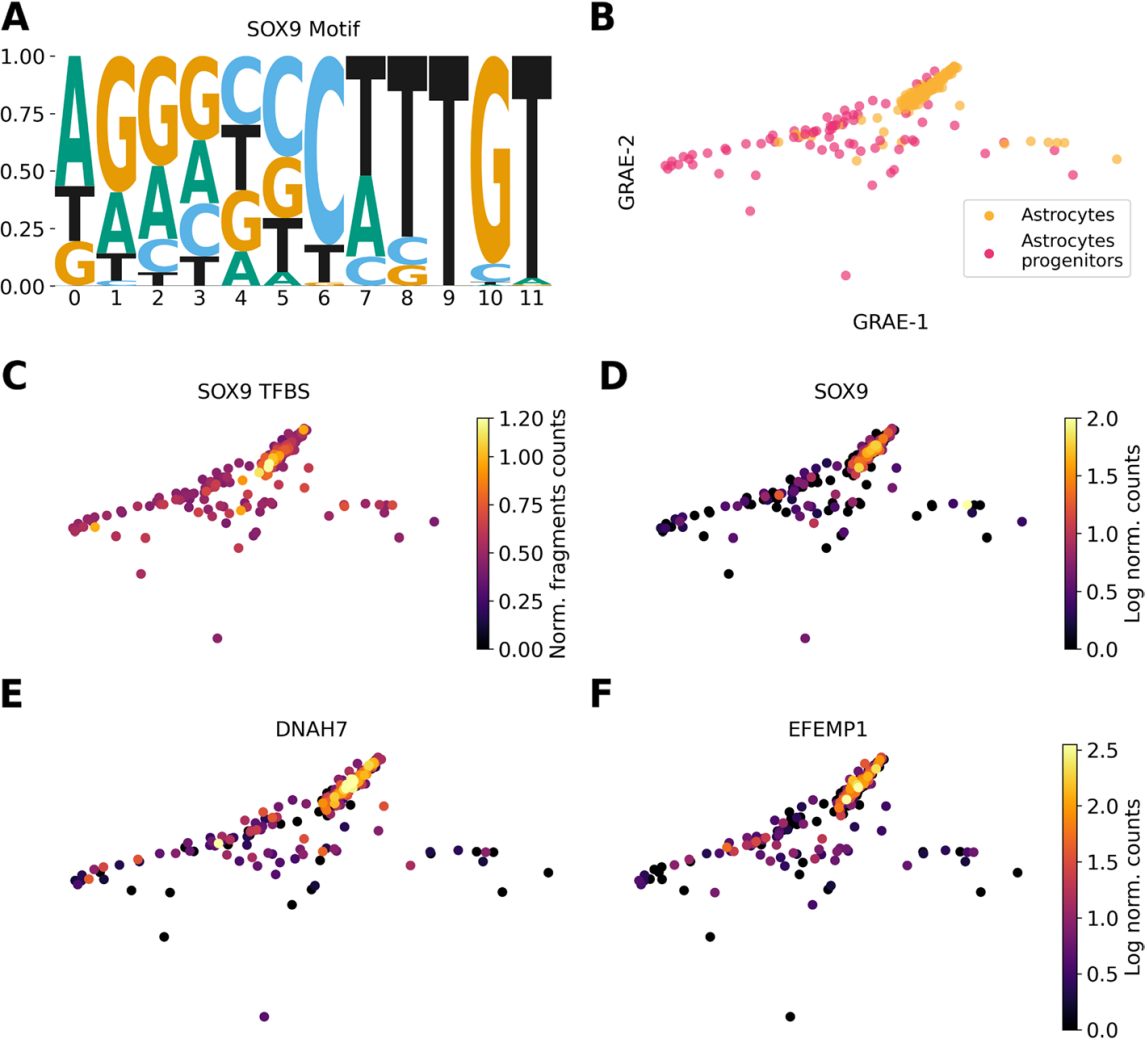
**A** *SOX9* motif. **B** GRAE embedding showing the differentiation from astrocytes progenitors to astrocytes. **C** Openness of *SOX9* TFBSs. **D**–**F** *SOX9*, *DNAH7* and *EFEMP1* expression

In the human brain data set [36], we explored both the GEX and ATAC modalities. Taking the scATAC-seq XAIF and running motif analysis, we identified several brain-specific motifs, which were not retrieved by motif analysis on the DA-specific features (Additional file 3 for the complete motif results). In the astrocytes progenitors, SEAGALL could identify motifs belonging to the well-known family of TFs SOX, such as *SOX9* (Fig. 6A), *SOX17* (Additional file 2: Fig. S6A) and *SOX1* (Additional file 2: Fig. S11B). *SOX9* is known to be essential for the correct development of astrocytes [37], and its promoter is activated to determine astrocyte differentiation [38]. Notably, the two-dimensional embedding obtained with GRAE can well capture the differentiation process from astrocytes progenitors to astrocytes along its horizontal axis (Fig. 6B). We measured the openness of all *SOX9* transcription factor binding sites (TFBSs) and it turns out that they are already open in the astrocyte progenitors with a maximal openness in astrocytes (Fig. 6C). On the other hand, the scRNA-seq modality shows that the expression of *SOX9* is very limited in the progenitors but very high in the mature cells (Fig. 6D). The other motif we retrieved is *SOX17* (Additional file 2: Fig. S11A), which is a TF known to be upregulated in astrocytes [39]. We discovered the openness of its TFBS as relevant for the identity of astrocyte progenitors; hence, we found a relevant TFBS openness in a progenitor population, which is related to the expression of the TF in the direct next cellular state. For both *SOX9* and *SOX17*, we therefore see the relevance of chromatin state priming the gene expression in progenitor cells, as suggested in [40]. Standard differential analysis could not highlight this dynamic behaviour. Focusing on GEX, SEAGALL ranked in the top fifty features of astrocytes the genes *DNAH7* and *EFEMP1*, which were not identified with standard differential analysis. The former is known to be expressed in intermediate astrocytes [41], and the latter is known to be expressed during synaptic development of astrocytes from iPSCs [42]. We correctly identified these genes during their positive gradient expression from the astrocytes progenitors to the astrocytes (Fig. 6E, F). In addition, reprogrammed astrocytes have been shown to express a *SOX1* positive state with neuronal stem cells characteristics [43], and we identified its motifs (Additional file 2: Fig. S11B) within the XAI features.

Also in the human brain data set, only SEAGALL was able to obtain the motifs of *JUNb*, *FOSL2* and *FOS* (Additional file 2: Fig. S11C–E) as enriched amongst the discovered XAIF for microglia in the ATAC modality. These TFs are known lineage-determining for microglia [44]. For GEX in microglia, only our method highlighted two important genes, *TLR2* and *RIPK2* (Additional file 2: Fig. S12A–C): the former modulates microglial activity [45], and the latter plays an essential role in the inflammatory response of microglia [46]. Last, in the brain cells annotated as inhibitory neurons, we found the motif of *ASCL1* (Additional file 2: Fig. S11F), which is known to specify and promote differentiation of GABAergic interneurons (i.e. inhibitory neurons) [47]. 

In the PBMC data set, *SOX4* is ranked amongst the most important genes in plasmacytoid dendritic cells (pDCs), antigen-presenting cells, but not amongst the differentially expressed ones (Additional file 2: Fig. S12D, E), and it is known to be involved in pDCs ontogeny [48]. In addition, we exclusively found *CR1* (Additional file 2: Fig. S12D, F) in the explanation of memory B cells, which is known to be necessary for the correct development of this cell type [49]. In the natural killers (NK) and T MAIT cells we uniquely retrieved, respectively, *LAIR2* and *CD8* (Additional file 2: Fig. S12D, G, H), which are their cell type markers [50, 51]. Combining the features discovered by SEAGALL with motif analysis and manual inspection, we show how SEAGALL can identify several relevant TFBS and genes which are known to be determinants of cell types and their lineages. These TF motifs and genes were not discovered by the classical differential analysis pipeline, showing that our method is able to extract meaningful biological insights which can contribute to the discovery of determinants of cell identity. In particular, we identified several features which were not identified using differential analysis, which related to the development and differentiation of cells, suggesting the ability of SEAGALL to capture features which are important in a dynamical state rather than only differences between populations.

## Discussion

In this study, we presented SEAGALL (Single-cell ExplAinable Geometry-Aware Graph Attention Learning pipeLine), a scalable (Additional file 2: Fig. S13) deep learning method based on manifold learning and explainable AI to analyse different modalities of single-cell data. SEAGALL combines a graph-regularised autoencoder (GRAE) and a graph attention network (GAT) with an explainable artificial intelligence (XAI) method to classify cells into cell types or states and to extract the most important input features for label prediction (Fig. 1). 

We applied SEAGALL to 10 single-cell data sets from three different omics (scRNA-seq, scATAC-seq, scChIP-seq) and showed that SEAGALL can consistently understand and explain cell identity from a perspective distinct from classical differential analysis. The combination of a manifold learning method and an autoencoder (GRAE) to reduce data dimensionality has been systematically applied to the single-cell field for the first time. We showed the impact of geometric regularisation through a deep ablation study. We replaced the GRAE with baseline methods, such as PCA and vanilla AE, as well as more advanced AEs (VAE, TAE, siVAE, scVI, PeakVI). The geometrical regularisation has revealed a winning strategy since it was able to reconstruct corrupted data with the highest success (Fig. 2) while robustly preserving the biological information about cell type on the task of building the cell-cell graph (Fig. 3). Therefore, this approach has revealed an effective and reliable method to build a cell-cell graph, which is the final representation of the input data. Moreover, using the geometry-aware graph as input to a classifier that applies an attention mechanism to increase the flexibility of the model, the classification performance reaches a maximum (Fig. 4). The main innovation of SEAGALL is the use of explainable AI to explore cell-type phenotypes, making our method highly translational. Often, deep learning focuses on predictive performance, retaining limited interpretability and preventing the direct gain of new biological knowledge. Here, we exploit a novel graph neural network explainer (GNNExplainer) to open the black box and extract specific, stable, and determinative features (Figs. 5 and 6) that drive cell type classification predictions. Thanks to its user-friendly code and tutorial, we made our method suitable and useful for real-world applications, since it can be directly applied to any count matrix from single-cell data. The deep learning method for learning the data sets ensures that the nonlinearity of the manifold, determined by the complex gene regulatory networks, is taken into account, whereas standard approaches based on PCA and DA do not. We applied SEAGALL to several single-cell data sets and showed that we are able to retrieve TFBSs which are driving factors of cell identity, but that have not been identified using a standard differential analysis pipeline (Fig. 6). Finally, SEAGALL can be applied to different single-cell data modalities, such as scATAC-seq, scRNA-seq and scChIP-seq data, reflecting the omic-independent hypothesis framework we proposed.

## Conclusions

SEAGALL lays the foundation for future geometry-aware models that can integrate multi-modal measurements, which are becoming increasingly common in the single-cell field. The model can be extended beyond predefined cell types, and in the future, it should be more thoroughly evaluated in its ability to learn any possible label. For that, it may be possible to use data sets of cell lines stimulated or infected with different agents to learn the driving features of the cellular response at each time point. The necessity of pre-defined cell labels prevents the model from discovering new cell states; however, any unsupervised clustering method can be used to cluster cells into related groups and SEAGALL can be applied to identify the molecular features that best describe the cluster labels. The suggested framework provides an explainable, geometry-aware method for single-cell analysis, helping to uncover new relevant regulatory features that define cell identity beyond differential analysis. The identification of chromatin priming states shows that SEAGALL can reveal the dynamic mechanisms underpinning cell fate decisions. This capability may have interesting implications for precision medicine, as the ability to pinpoint specific transcription factors and genes that drive cell states in complex tissues could suggest novel therapeutic targets for diseases.

## Methods

### Single-cell RNA-seq data processing

Single-cell RNA-seq quantifies the abundance of RNA molecules, mainly mRNA, within a cell. For each single-cell the sequencer reads the transcripts that belong to it; hence, the output is a raw set of reads which need to be aligned and quantified. For the two human multi-ome data sets (PBMC [29] and brain [36]), raw reads were processed using Cell Ranger Arc 2.0.2, aligning them to the complete human genome (T2T) [52]. The GEX count matrix for the HSPC data set has been downloaded from [53]. The GEX count matrix of the mouse brain data set [54] has been taken from 10X website[50]. We did not impute missing values. We computed the probability distributions across cells of the number of non-zero genes and the number of mitochondrial reads; we filtered out all cells with a value for either variable outside the 5% or 95% quantiles of their distributions. Similarly, genes present in the lower or upper 5% quantile of the cells were removed. Data were library-size normalised. We kept the top 10% highly variable genes. Lastly, the data were log-transformed. Differentially expressed genes (DEG) between cell types were calculated using the Wilcoxon test with Scanpy [7]. We kept the fifty most differentially expressed genes for our analysis.

### Single-cell ATAC-seq data processing

Single-cell ATAC-seq is a popular technique to profile chromatin openness at the single-cell level. Typically, when analysing scATAC-seq data, the measurements are summarised in a count matrix based on the positions of signal enrichment in the genome, called peaks [55]. To construct a count matrix, peaks are called on the pseudo-bulk signal and for each cell and each peak is counted the number of reads that fall into each peak. The matrix structure is identical to scRNA-seq, but in the latter case, the features are transcripts. The reads of the kidney data set [56] have been processed using Cell Ranger ATAC 2.1.0 [57] and the ones of the PBMC and human brain data sets have been aligned with Cell Ranger Arc 2.0.2; in both cases, the reference genome is the T2T human genome [52]. Count matrices were built using Episcanpy [13] from the fragments and peak files obtained with MACS2 [58]. We did not impute missing values. We computed the distribution of the number of features per cell and we filtered out cells having a number of features lower than the 5% quantile or higher than the 95% quantile of this distribution. Cells with a transcription start site (TSS) enrichment score lower than 2 and a nucleosome signal higher than 2 have been filtered out. Features (peaks) present in less than 5% or more than the 95% quantile of the cells have been removed. Data were library-size normalised. Only peaks with a variance above the 80% quantile of the variance distribution were kept, with a maximum of 30000 features. This number of peaks represents about 10–20% of the initial peaks, which is the same ratio of highly variable genes retained in scRNA-seq data sets in the literature, i.e., about 3000-5000 out of the about 30000 that are profiled. Lastly, the data were log-transformed. For the mouse data set, we downloaded the fragments file from 10X database. The fragments file of the HSPC data set was downloaded from the original publication [53]. Before building the count matrix and filtering, we called peaks using MACS [58] following the procedure described above. scChIP-seq experiment count matrices were downloaded from the original publication [59]. In this case, the features are windows of constant size (50kb) spanning the whole genome. We processed the data as peaks since the processing does not rely on any peak-specific assumption. Differential open peaks or windows between cell types are calculated with the Wilcoxon test. We kept the fifty most differentially open peaks or windows for our analysis. The choice of these parameters treats each data set fairly: since the thresholds are based on quantiles of distributions, we always impose the same rigidity on the quality control fitting the intrinsic properties of the data sets, such as sparsity and sequencing depth.

### Cell type annotation

For the HSPC [53], kidney [56] and breast cancer [59] data the cell type annotation is provided from the authors. The cell type annotation of the mouse brain is taken from [53] and it is based on marker genes. The human PBMC data set has been manually annotated following the muon tutorial [60]. The mouse brain has been manually annotated with marker genes and the procedure is shown in our GitHub. Each data set consists of a different number of cell types (Additional file 1: Tables S1 and S2).

### Embedding and graph construction

Once the count matrices are cleaned, we use GRAE to build the cell-cell graph. First, PHATE is applied as a manifold learning method; it can capture both global and local structure of the data and embed it into a lower-dimensional representation of arbitrary dimension. The loss function of the autoencoder, which is the mean squared error (MSE) between original and reconstructed space, is then regularised by adding a term which increases if the latent space differs more from the PHATE embedding. In other words, the total loss function *L* is composed of two terms: a reconstruction term $L_{r}$ and a regularisation term

1 $$\begin{aligned} L(X, E) = L_{r}(X, f^{-1}(f(X))) + \lambda L_{g}(f(X), \Xi ) = MSE(X, f^{-1}(f(X))) + \lambda \sum \limits _{i=1}^{N} {|| \xi _{i}-f(x_{i}) ||^2} \end{aligned}$$

where *X* is a set of *N* data points such that $x_{i} \in \textrm{I}\!\textrm{R}^{\textrm{d}}$, $\Xi$ is the PHATE embedding of *X* such that $\xi _{i} \in \textrm{I}\!\textrm{R}^{\textrm{p}}$ with $p<< d$, *f* and $f^{-1}$ are, respectively, the encoding and decoding function. The dimension of the latent representation varies for each count matrix to fit the data set complexity and it is set as the cubic root of the number of features. Within the latent space, pairwise Euclidean distance between cells is computed and then a k-NN graph is built, with $k=15$, since this is the standard value according to the Scanpy tutorial that we took as reference [7]. The k-NN graph had already been used in the literature as an input graph for GNNs [61], but there is also a technical motivation that led us to a constant degree network: building a correlation-based or distance-based graph is intrinsically problematic; after computing pairwise distances or correlations, a cut-off is applied to the maximum distance or minimum correlation. Each node may have any number of neighbours in the interval $[0, N-1]$. We tested this possibility and it turns out the resulting graph is extremely dense (Additional file 2: Fig. S14), which may lead to nonsensical connections and makes the training of the GNNs extremely time and energy demanding. The graph is the final representation of the data set, which contains the connectivity pattern and the geometry of the input manifold.

### Cell type classification with GNNs

Graph neural networks are a type of neural network that can process data with a graph structure. GNNs take as input a graph $G = (V, E)$, where $V \in \mathbb {N}$ is the set of nodes and $E \subseteq V \times V$ is a set of edges, also known as links, between nodes. Each node can have a feature vector that defines the properties of the nodes. In our context, the feature vector is the gene expression or the chromatin openness vector. From a point cloud perspective, the embedding of each point is a function of the point itself and the points close to it. Let $G = (V, E)$ be an undirected graph containing *N* vertices, $x_{i} \in {\textrm{I}\!\textrm{R}}^d$ is the initial representation of node *i*, $\mathcal {N}_{i} = \{j \in V \vert (j,i) \in E\}$ the neighbours of node *i*, then the first layer of the GNN will create a new representation of the node $x_{i}'$ according to

2 $$\begin{aligned} {x}_i^{\prime } = \gamma _{\mathbf {\Theta }} \left( \textbf{x}_i, \bigoplus _{j \in \mathcal {N}_{i}} \, \phi _{\mathbf {\Theta }} \left( \textbf{x}_i, \textbf{x}_j,\textbf{e}_{j,i}\right) \right) \end{aligned}$$

GNNs are a broad class of neural networks which rely on Eq. 2, known as the message passing equation [26]. We decided to apply a more refined version of the base message-passing layer called graph attention networks (GATs) [19], which applies an attention mechanism to the embedding function. The embedding of the nodes follows

3 $$\begin{aligned} \textbf{x}^{\prime }_i = \sum \limits _{j \in \mathcal {N}_{i} \cup \{ i \}} \alpha _{i,j} \mathbf {\Theta }_t\textbf{x}_{j} \end{aligned}$$

where $\alpha _{i,j}$ are the attention coefficient and they are in the form of

4 $$\begin{aligned} \alpha _{i,j}=\frac{\exp \left( \textbf{a}^{\top }\textrm{LeakyReLU}\left( \mathbf {\Theta }_{s}\textbf{x}_i +\mathbf {\Theta }_{t} \textbf{x}_j\right) \right) }{\sum \nolimits _{k \in \mathcal {N}(i) \cup \{ i \}}\exp \left( \textbf{a}^{\top }\textrm{LeakyReLU}\left( \mathbf {\Theta }_{s} \textbf{x}_i +\mathbf {\Theta }_{t} \textbf{x}_k\right) \right) } \end{aligned}$$

where $\mathbf {\Theta } \in \textrm{I}\!\textrm{R}^{\textrm{dxd}^{'}}, \textbf{a} \in \textrm{I}\!\textrm{R}^{2\textrm{d}^{'}}$ are learned parameters, $\bigoplus$ is any differentiable and permutation invariant function such as sum or mean, and $\gamma _{\mathbf {\Theta }}$ and $\phi _{\mathbf {\Theta }}$ are differentiable functions such as MLPs. $\alpha _{i,j}$ are related to edges and allow for a dynamic understanding of the importance of the links, making sure that the model does not get misled by spurious ones. It is important to notice that the importance mechanism refines the graph connectivity; therefore, it does not act on the features of the nodes but on their connections. Thus, the key property of GNNs is the ability to create latent representations of a local neighbourhood rather than a single point. The rationale for choosing GNNs relies on this property: we want to have a local analysis of each cell, aiming for a local ensemble study, rather than treating them totally independently. Each count matrix with its own graph is given as input to the GNN classifier; the target output is the cell type of each node, which is defined as described in Cell type annotation section. Our specific model consists of a graph neural network with two layers, the first one to create a latent representation of the input and the second one to perform the classification task. The dimension of each layer is defined with hyperparameter optimisation (HPO) [62] case by case. The model is trained with Adam [63] optimiser with learning rate and weight decay estimated with HPO.

### GAT explanation

Once the model is trained, an XAI method is applied to it. We choose to apply “GNNExplainer” [21] which is a model-agnostic method. It creates a graph and a feature mask to spot the minimum set of features and edges of each node sufficient to predict the class. We assume that the nature of our data defines a real function *f* that labels objects, nodes in the case of GNNs, representing cells in our context. The GNN model $\Phi$ receives as input a graph *G* and a feature vector *X* as explained in the previous paragraph. In practice, $\Phi$ learns a probability $P_{\Phi }(Y|G, X)$ with *Y* random variable for the classes $\{c_{i}\} \mid i=1,..,C$ representing the probability of nodes to belong to each of the classes. After the training, the model is fixed and it will be used to make predictions. The crucial point of the explainer is the fact that each node has a computation graph *G* and certain node features *X* that completely determine all the information that are necessary to predict $\widehat{y}$ at certain node *v*. Given a node $v_{i}$ the explainer finds the sub-graph $G_{s} \subseteq G$ and the associated features $X_{s} = \{x_{j} | v_{j} \in G_{s}\}$ that maximise the probability of having seen the prediction $\widehat{y} = \Phi (G_{s}, X_{s})$ where $\Phi$ is the trained GNN. Indicating as *MI* the mutual information function and *H* the entropy function, the GNNExplainer solves the following problem

5 $$\begin{aligned} max_{\{G_{s}\}} MI(Y, (G_{s}, X_{s})) = H(Y) - H(Y|G=G_{s}, X=X_{s}) \end{aligned}$$

MI quantifies the variation in the prediction probability when the graph and the features are $G_{s}$ and $X_{s}$ instead of *G* and *X*, with the feature vector constrained to be much smaller than the original one. In practice, for each node we obtain the features ranked by their importance. Since we are interested in the cell types explanations, we average the feature importance of all the nodes belonging to the same class to obtain the most relevant features for each label.

### AE models

Whereas GRAE [5], PeakVI [12] and scVI [11], siVAE [28] are released as packages, we had to implement the models for topological, vanilla AE and variational AE. The latter three methods are based on the same architecture, which consists of one input layer, one hidden layer with dimension equal to the square root of the input size, and a latent layer with dimension equal to the cubic root of the input size. Variable layer sizes are important to account for the complexity of the data set. The best values of dropout, learning rate, weight decay, the weight of topological regularisation, and the signature of the p-norm (the latter only for TAE) have been estimated using HPO implemented with the Optuna package. Each HPO consists of 25 runs to explore the parameter space within defined intervals (Additional file 1: Table S8). We applied annealing to the KL-divergence weight in the VAE. We used a subset of count matrices to explore the HPO and we then applied the same parameters to each matrix.

### Model benchmarking

We carried out a breakdown of SEAGALL, testing each of its main parts: the embedding method (GRAE), the classifier (GAT) and the explainer (GNNExplainer). We used six count matrices for benchmarking the embedding method and the classifier, two from scRNA-seq and four from scATAC-seq (Additional file 1: Tables S1 and S2 for the cell type composition, dimensions and links to raw data). They are two multi-modal data sets for which the scRNA-seq and the scATAC-seq were treated separately (human brain and human PBMC), the scATAC-seq part of a multi-modal data set of mouse embryonic brain, and a scATAC-seq data set of kidney [56] (see Methods for the count matrix construction and processing). We tested the GRAE together with a topological autoencoder (TAE) [27], a standard autoencoder (AE), a variational one (VAE), PeakVI [12], scVI [11], siVAE [28] and linear PCA. The TAE was included to compare the GRAE to an AE with a similar rationale behind: while the GRAE regularises the loss function considering that the geometry of the data should be preserved in the latent space, the TAE preserves the topology of the input space by applying persistent homology. Geometry is a more specific and local property than topology; however, neither GRAE nor TAE make assumptions about the data sets, which makes them applicable to, in principle, any kind of biological data. The VAE and the AE are used as baseline autoencoder models to compare sophisticated methods with simpler ones. scVI and PeakVI are state-of-the-art methods for modelling scRNA-seq and scATAC-seq data, respectively. siVAE is an interpretable variational AE meant to analyse genomic data. PCA is included to quantify the difference between linear and nonlinear methods. We tested the ability of the embedding methods (see next paragraph) to recover the original data after adding artificial dropout and the quality of the cell-cell graph computed in the different latent spaces. To quantify the latter feature, we measure the homogeneity of the cell-cell graph in terms of cell type composition of the neighbourhood and the performance of a GNN classifier varying the input graph. We then tested the GAT and also a Graph Convolutional Network (GCN) architecture, computing F1-score, accuracy, precision and recall of the classifiers. Last, we measured the stability and the specificity of the GNNExplainer. We also measured the classification and explanation performance of the final model on a scChIP-seq data set of breast cancer (Additional file 1: Tables S1 and S2), in which H3K27me3 was measured at the single-cell level [59].

### Input data reconstruction

To test the robustness of the AEs, we measured their ability to reconstruct the input data after corruption. To corrupt the data, we applied increasing dropout from 10% to 50% of the features in 10% increments to each count matrix, and trained each model on the corrupted data. When applying dropout, the choice of which features to remove is random; therefore, it may happen that we remove features that are particularly important for one model but not for another. To ensure our results are not biased by this factor, we repeat the experiment 10 times, varying the features to drop out at each level. All the models have been trained with the same patience (30) and maximum number of epochs (300). We used 85% of the data for training and 15% for validation. For each run, each level of dropout, and each model, we measured the MSE and the Spearman correlation coefficient between the original data (the uncorrupted one) and the model-reconstructed data. We then computed the average MSE and Spearman rank correlation for each level and model across the ten runs. To exclude outliers from the MSE computation, we applied the metrics “MSE1obs” and “MSE1imp” as suggested in [30]. The former is the MSE computed only considering the top 1% most covered features, where the coverage is computed from the original (observed) count matrix. The latter follows the same logic, but the coverage is calculated using the reconstructed (imputed) count matrix [30]. Reconstruction performance was also quantified using the Negative Binomial (NB) loss, defined as the negative log-likelihood (NBloss in the figures) of the observed count data under a negative binomial noise model. This formulation accounts for the discrete and overdispersed nature of single-cell measurements and provides a more robust alternative to mean squared error for comparing autoencoder reconstructions [11].

### Graph homogeneity

We assumed that a good latent space leads to a k-NN graph where neighbours of a node belong to the same cell type. The more homogeneous the neighbourhood, the more the AE can locate cells close to each other in the latent space, sharing the same biological functions. After applying each dimensionality reduction method described in the previous paragraph (GRAE, TAE, AE, VAE, PeakVI, scVI, siVAE, and PCA) without dropout to each count matrix, we computed the k-NN graph (k=15) from their latent spaces. For each cell we computed how many different cell types are found in its neighbourhood. We divided this value by both the number of neighbours (15) and the number of cell types (varying across data sets) to obtain a heterogeneity score. Lastly, we computed heterogeneity as one minus heterogeneity. We average the values over the fifty runs of each embedding method.

### Classification and XAI experiments

To test the quality of each embedding method, we used their latent space to build the cell-cell k-NN graphs (k=15) and we gave the graphs as input to a graph neural network node classifier. We tested the combination of dimensionality reduction methods (GRAE, TAE, AE, VAE, PeakVI, scVI, siVAE, and PCA) with two GNNs: GAT and GCN (see the Cell type classification with GNNs paragraph for details on the models). We run each combination fifty times. Each training run started with a different random seed to ensure the models did not always start from the same point in the parameter space. Both GAT and GCN are trained for 300 epochs with a patience of 30 epochs. The data sets have been split into training, validation, and test sets with ratios of 70%, 10%, and 20%, respectively. Before training the classifier, we run a 25-step HPO study to select the best values (Additional file 2: Fig. S15) of each hyperparameter of the GNNs, within defined ranges (Additional file 1: Table S9). After each training we applied the explainer for 300 epochs and saved the fifty most important features for each label, i.e. for each cell type. Accuracy, precision, recall and F1 score have been computed in the standard way using Scikit-learn [64, 65]; the specificity of the explainer is defined as one minus the average intersection of the top fifty most relevant features across cell types. Stability is defined as the average intersection of the explanation for the same cell type across the fifty runs of the classifier and the explainer. Feature coverage is defined as the number of cells in which the feature has been detected, i.e., it has a non-zero value. The expression, or openness, is defined as the number of reads mapping to that feature. The evaluation of the scalability has been run on an Intel® Xeon® Processor E5-4660 v3 12 cores, 1.9 GHz using 12 cores and 700GB of RAM. We incrementally sampled cells from a 1.3-million-cell data set [66] of the CELLxGENE project [67, 68] (Additional file 1: Tables S1 and S2). We measured the runtime of the model and the actual RAM usage, which does not take into account the memory that is needed to store the data but it is the RAM increment when running the tool.

## Supplementary Information

Additional file 1. Supplementary tables.

Additional file 2. Supplementary figures.

Additional file 3. Complete list of motifs identified with HOMER.

Additional file 4. Issues encountered during the attempted usage of competitor computational methods, particularly concerning their installation and operation.

## Data Availability

Code and tutorial for SEAGALL [69] are available at https://github.com/gmalagol10/seagall under GPL3 license. All the links to download the data sets supporting the conclusions of this article are available in Additional file 1: Tables S1 and S2. The code to reproduce all the results is available at https://github.com/gmalagol10/seagall/tree/main/reproducibility and at Zenodo [70] under GPL3 license. The HSPC [53], Kidney [56], breast cancer [59] and ageing [66] data sets are available at, respectively, GSE209878 [71], GSE172008 [72], GSE117309 [73], GSE299043 [74]. The human brain data set [36] is available at https://www.10xgenomics.com/datasets/frozen-human-healthy-brain-tissue-3-k-1-standard-2-0-0, the PBMC [29] is available at https://www.10xgenomics.com/datasets/pbmc-from-a-healthy-donor-no-cell-sorting-10-k-1-standard-2-0-0 and the mouse brain [54] is available at https://www.10xgenomics.com/datasets/fresh-embryonic-e-18-mouse-brain-5-k-1-standard-1-0-0.
