# Supplementary material for: Geometry-aware graph attention networks to explain single-cell chromatin states and gene expression with SEAGALL
Source: Genome Biol. 2026 Apr 23;27:188. doi: 10.1186/s13059-026-04066-2 (PMC13238118; doi:10.1186/s13059-026-04066-2)
Supplement: Supplementary file 1 — Additional file 1. Supplementary tables. [file 13059_2026_4066_MOESM1_ESM.pdf]

Human brain	Breast cancer	PBMC	HSPC	Mouse brain	Kidney	Aging
Multiome	scChIP-seq	Multiome	Multiome	Multiome	scATAC-seq	scRNA-seq
Molec_Layer_Interneur	HBCx-95	Monocytes_CD16	HSC	Subplate	Endo	b_age
Microglia	HBCx-95-CapaR	T_CD8_naive	LMPP	Deeper_Layer	PT	b_gc
Oligodendrocyte	HBCx-22	B_naive	MPP	Upper_Layer	CDPC	b_memory
Purkinje_neuron_layer	HBCx-22-TamR	B_memory	MEP	IPC	Immune	b_naive
Inhibitory_neuron_PVALB_SST		NK	Prog_B	V_SVZ	LOH	cd3_gd
Purkinje_neuron_ITPR1		T_MAIT	Prog_DC	RG_Astro_OPC	CDIC	cd4_cm
Inhibitory_neuron		T_CD4_naïve	Erythrocyte	Ependymal_cells	Podo	cd4_naive
Purkinje_neuron_FOXP2		DCm	GMP		DCT	cd4_tem
Astrocyte		Monocytes	Granulocyte			cd4_temra
Inhibitory_neuron_MAF		T_CD4_memory	Prog_MK			cd4_treg
Astrocyte_progenitor		T_CD8_activated	Platelet			cd4_trm
		DCp				cd8_cm
						cd8_mait
						cd8_naive
						cd8_tem
						cd8_temra
						cd8_trm
						dc1
						dc2
						dc_migratory
						ilc1
						ilc3
						macrophage
						mast_cell
						monocyte_classical
						monocyte_nonclassical
						nk_cd56br
						nk_cd56dim
						nk_ilc_precursor
						pdc
						plasma_cell
						plasmablast
						progenitor

Table S1: Cell type composition and links to the original raw data for each dataset.

Dataset	Feature space	# Features	# Cells	GEO	DOI/URL
HSPC (Multiome)	GEX	2689	10389	GSE209878	10.1038/s41587-022-01476-y
HSPC (Multiome)	Peaks	30000	10350	GSE209878	10.1038/s41587-022-01476-y
Kidney (scATAC-seq)	Peaks	25901	4624	GSE172008	10.1038/s41588-021-00909-9
Human brain (Multiome)	GEX	3003	2441	Not available	https://www.10xgenomics.com/datasets/frozen-human-healthy-brain-tissue-3-k-1-standard-2-0-0
Human brain (Multiome)	Peaks	17886	2726	Not available	https://www.10xgenomics.com/datasets/frozen-human-healthy-brain-tissue-3-k-1-standard-2-0-0
PBMC (Multiome)	GEX	2761	5700	Not available	https://www.10xgenomics.com/datasets/pbmc-from-a-healthy-donor-no-cell-sorting-10-k-1-standard-2-0-0
PBMC (Multiome)	Peaks	12781	6372	Not available	https://www.10xgenomics.com/datasets/pbmc-from-a-healthy-donor-no-cell-sorting-10-k-1-standard-2-0-0
Mouse brain (Multiome)	GEX	2237	2721	Not available	https://www.10xgenomics.com/datasets/fresh-embryonic-e-18-mouse-brain-5-k-1-standard-1-0-0
Mouse brain (Multiome)	Peaks	29114	3027	Not available	https://www.10xgenomics.com/datasets/fresh-embryonic-e-18-mouse-brain-5-k-1-standard-1-0-0
Breast cancer (scChIP-seq)	Windows	10908	4636	GSE117309	10.1038/s41588-019-0424-9
Aging	GEX	35475	1281499	GSE299043	10.1038/s41590-025-02241-4

Table S2: Number of features and number of cells for each count matrix.

	Kidney Peaks	Human brain Peaks	Human brain GEX	Mouse brain Peaks	PBMC Peaks	PBMC GEX
GRAE	**0.9907±0.0001**	**0.9911±0.0001**	**0.9917±0.0001**	**0.9849±0.0002**	**0.9909±0.0001**	**0.9922±0.0001**
TAE	0.9783±0.0002	0.9763±0.0002	0.9815±0.0003	0.9676±0.0002	0.9808±0.0002	0.9768±0.0001
AE	0.9698±0.0002	0.9661±0.0003	0.9814±0.0003	0.9594±0.0003	0.9725±0.0001	0.9750±0.0001
VAE	0.9630±0.0002	0.9664±0.0002	0.9664±0.0002	0.9569±0.0003	0.9670±0.0001	0.9672±0.0001
PeakVI	0.9637±0.0001	0.9669±0.0002		0.9579±0.0002	0.9676±0.0001	
scVI			0.9783±0.0002			0.9723±0.0001
siVAE	0.9749±0.0002	0.9704±0.0002	0.9809±0.0002	0.9652±0.0002	0.9786±0.0001	0.9750±0.0001
PCA	0.9767±0.0001	0.9771±0.0002	0.9921±0.0001	0.9701±0.0002	0.9856±0.0001	**0.9923±0.0001**

Table S3: Average homogeneity of the k-NN graphs computed from the different data representations. Each entry reports the mean value and three times the uncertainty on the mean. Bold font highlights the best-performing method(s). Empty cells indicate invalid combinations of modality and method (e.g., PeakVI with GEX data, scVI with scATAC-seq data).

	**Accuracy**	**F1M**	**F1W**	**Precision**	**Recall**	**Specificity**	**Stability**
GAT	0.929 ± 0.006	0.89 ± 0.01	0.928 ± 0.006	0.931 ± 0.006	0.929 ± 0.006	0.974 ± 0.004	0.91 ± 0.03
GCN	0.933 ± 0.006	0.89 ± 0.01	0.932 ± 0.006	0.935 ± 0.006	0.933 ± 0.006	0.974 ± 0.004	0.91 ± 0.02

Table S4: Classification performance of the two GNN architectures: GAT and GCN. Each entry reports the mean value and three times the uncertainty on the mean.

	Accuracy	F1M	Precision	Recall	Specificity	Stability
GRAE	**0.929±0.006**	**0.89±0.01**	**0.931±0.006**	**0.929±0.006**	0.974±0.004	**0.91±0.03**
TAE	0.86±0.01	0.79±0.02	0.86±0.01	0.86±0.01	**0.975±0.004**	**0.91±0.03**
AE	0.66±0.04	0.56±0.04	0.70±0.03	0.66±0.04	0.970±0.005	0.82±0.04
VAE	0.37±0.02	0.33±0.03	0.47±0.03	0.37±0.02	0.957±0.006	0.80±0.04
PeakVI	0.916±0.006	0.85±0.01	**0.919±0.006**	**0.916±0.006**	**0.980±0.000**	**0.91±0.03**
scVI	**0.938±0.006**	**0.89±0.01**	**0.940±0.006**	**0.938±0.006**	**0.977±0.003**	0.89±0.03
siVAE	0.59±0.03	0.47±0.03	0.631±0.026	0.59±0.03	0.972±0.004	0.81±0.04
PCA	0.73±0.03	0.67±0.03	0.78±0.02	0.73±0.03	**0.980±0.001**	0.84±0.04

Table S5: Classification performance obtained using the different data representations. Each entry reports the mean value and three times the uncertainty on the mean. Bold font highlights the best-performing method(s). Underlined font highlights the second-best-performing method(s).

	Accuracy	F1M	Precision	Recall	Specificity	Stability
GRAE	**0.919±0.008**	**0.863±0.01**	**0.922±0.008**	**0.92±0.01**	0.969±0.006	**0.92±0.04**
TAE	0.83±0.01	0.73±0.01	0.84±0.01	0.83±0.01	0.979±0.002	**0.91±0.04**
AE	0.60±0.04	0.48±0.05	0.65±0.04	0.60±0.04	0.962±0.008	0.79±0.05
VAE	0.30±0.02	0.23±0.02	0.39±0.02	0.30±0.02	0.952±0.010	0.77±0.04
PeakVI	**0.91±0.01**	0.84±0.01	**0.909±0.007**	**0.91±0.01**	**0.981±0.001**	0.89±0.05
siVAE	0.54±0.02	0.37±0.02	0.58±0.02	0.55±0.02	0.967±0.006	0.77±0.06
PCA	0.63±0.03	0.54±0.02	0.70±0.01	0.63±0.03	**0.979±0.002**	0.79±0.05

Table S6: Classification performance obtained using different representations of the scATAC-seq data. Each entry reports the mean value and three times the uncertainty on the mean.

	Accuracy	F1M	Precision	Recall	Specificity	Stability
GRAE	**0.95±0.01**	**0.929±0.007**	**0.950±0.009**	**0.95±0.01**	0.9819±0.0007	0.89±0.04
TAE	0.91±0.02	0.87±0.02	0.92±0.02	0.91±0.02	0.97±0.01	**0.90±0.03**
AE	0.78±0.05	0.72±0.04	0.80±0.04	0.78±0.05	**0.9833±0.0006**	0.87±0.04
VAE	0.50±0.03	0.53±0.02	0.67±0.02	0.50±0.03	0.9660±0.0007	0.87±0.04
scVI	**0.956±0.006**	**0.928±0.005**	**0.959±0.006**	**0.956±0.006**	0.975±0.007	**0.92±0.02**
siVAE	0.67±0.06	0.66±0.03	0.73±0.05	0.67±0.06	0.9792±0.0006	0.88±0.04
PCA	**0.950±0.007**	**0.921±0.006**	**0.953±0.007**	**0.950±0.007**	**0.9822±0.0006**	**0.92±0.02**

Table S7: Classification performance obtained using different representations of the scRNA-seq data. Each entry reports the mean value and three times the uncertainty on the mean. Bold font highlights the best-performing method(s). Underlined font highlights the second-best-performing method(s).

	Regularisation weight	P for the P-norm	Weight decay	Learning rate	Dropout
Min value	1	1	0.0001	0.0001	0.1
Max value	30	5	0.1	0.1	0.8
Models	AE	TAE	TAE, AE, VAE	TAE, AE, VAE	TAE, AE, VAE

Table S8: Minimum and maximum values explored during hyperparameter optimization of the autoencoder models.

	Hidden dimension	Number of heads	Weight decay	Learning rate
Min value	32	4	0.0001	0.0001
Max value	256	12	0.1	0.1

Table S9: Minimum and maximum values explored during hyperparameter optimization of the GNN models.
