# Supplementary material for: Geometry-aware graph attention networks to explain single-cell chromatin states and gene expression with SEAGALL
Source: Genome Biol. 2026 Apr 23;27:188. doi: 10.1186/s13059-026-04066-2 (PMC13238118; doi:10.1186/s13059-026-04066-2)
Supplement: Supplementary file 2 — Additional file 2. Supplementary figures. [file 13059_2026_4066_MOESM2_ESM.pdf]

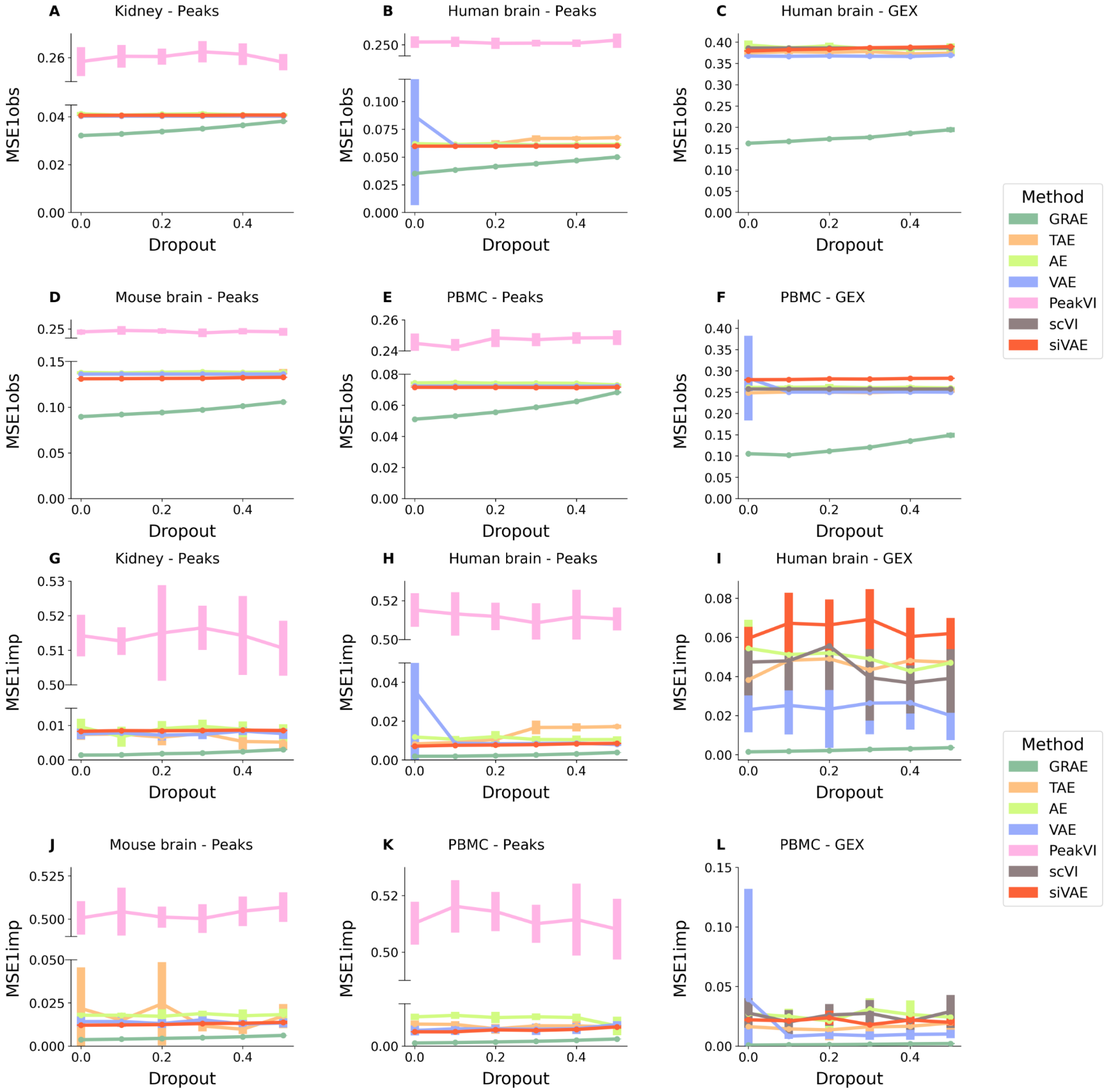

Fig. S1: **A**-**F** average MSE on the top 1% observed covered features at different levels of artificial dropout for each AE. **G**-**L** average MSE on the top 1% imputed covered features at different levels of artificial dropout for each AE. Each point is the average of ten runs, and the height of the error bar represents three times the uncertainty on the mean.

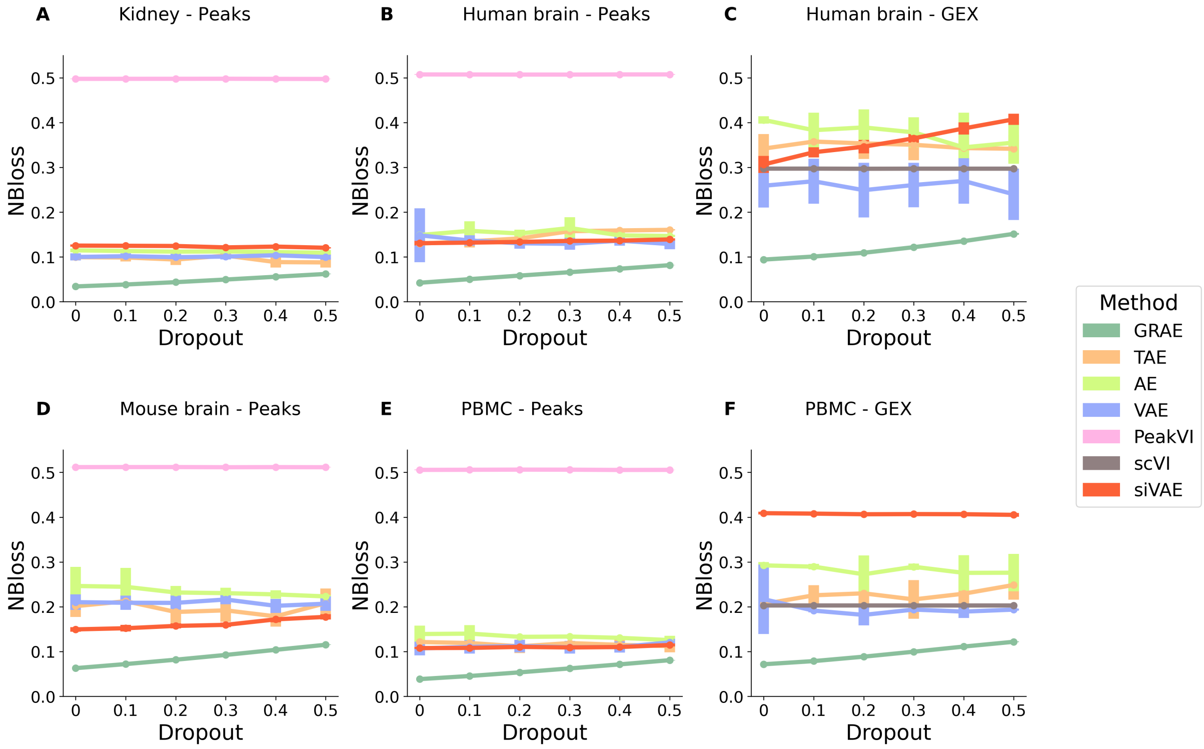

Fig. S2: **A**-**F** average negative binomial loss between input and reconstructed output at different levels of artificial dropout for each AE. Each point is the average of ten runs, and the height of the error bar represents three times the uncertainty on the mean.

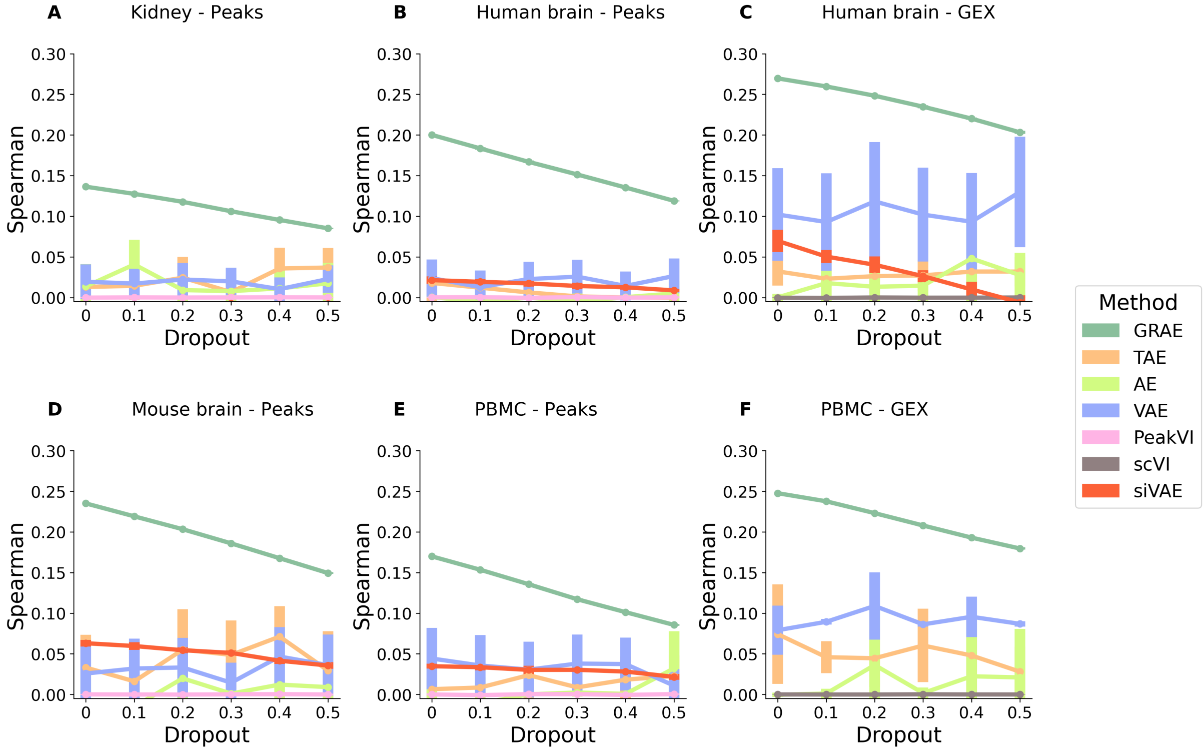

Fig. S3: **A**-**F** average Spearman correlation between input and reconstructed output at different levels of artificial dropout for each AE. Each point is the average of ten runs, and the height of the error bar represents three times the uncertainty on the mean.

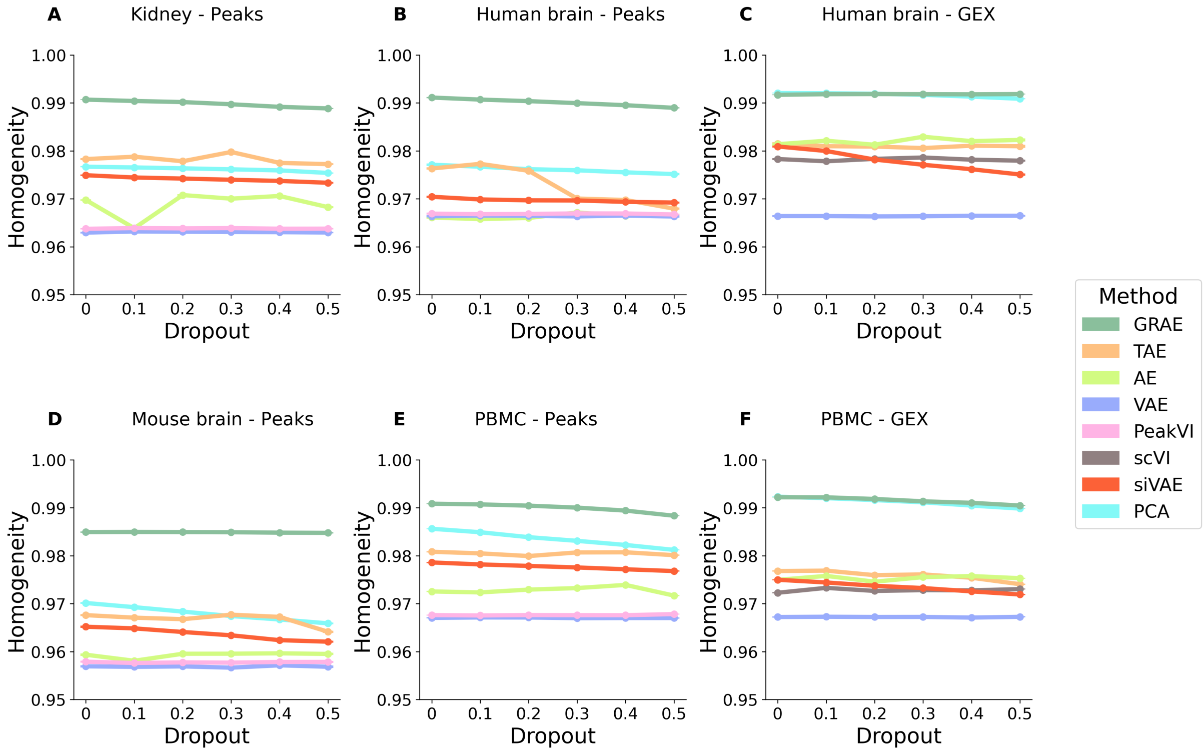

Fig. S4: **A**-**F** average homogeneity of the cell-cell graph at different levels of artificial dropout for each AE. Each point is the average of ten runs, and the height of the error bar represents three times the uncertainty on the mean.

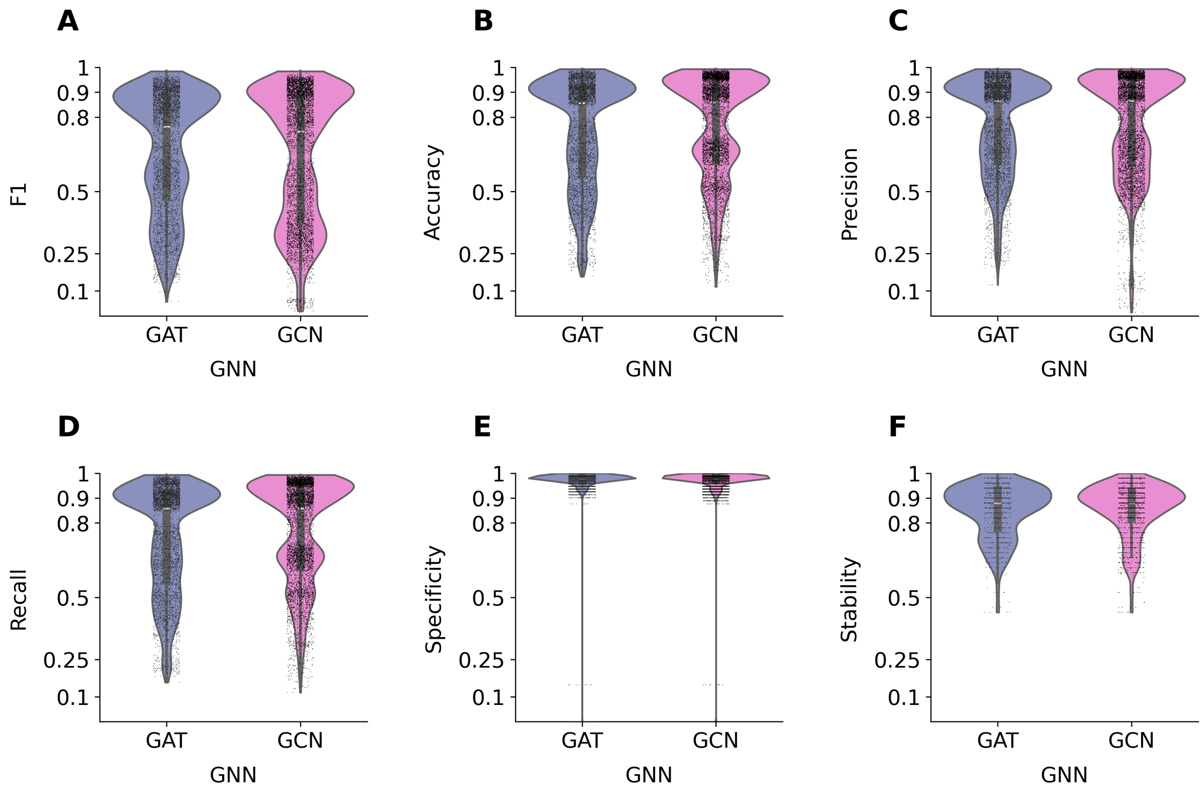

Fig. S5: **A**-**D** classification performances of the two GNN architectures. **E**-**F** specificity (left) and stability (right) of the two GNN architectures.

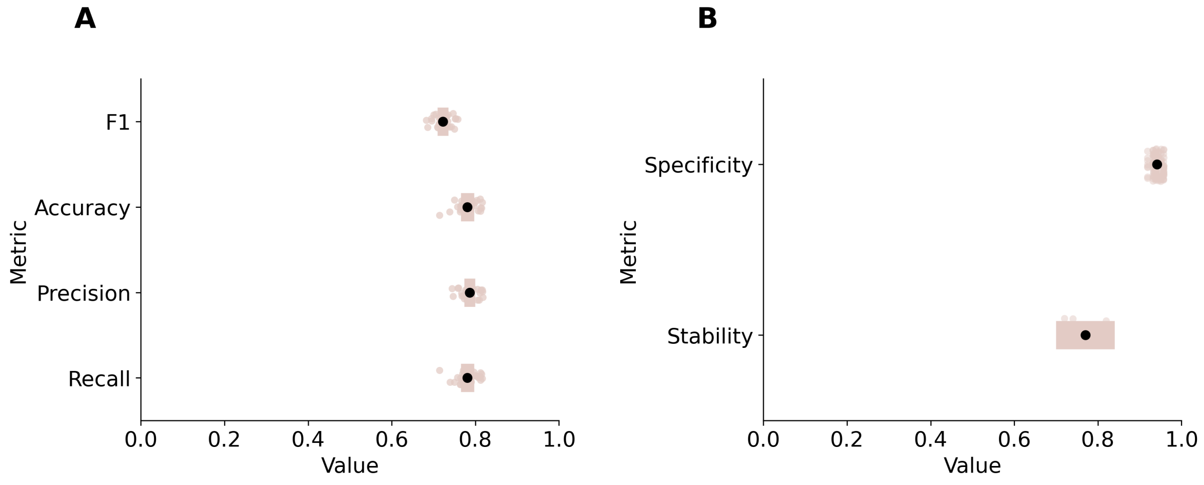

Fig. S6: **A** classification performance of the final model on the scChIP-seq. **B** stability and specificity of the explainer on the scChIP-seq data set.

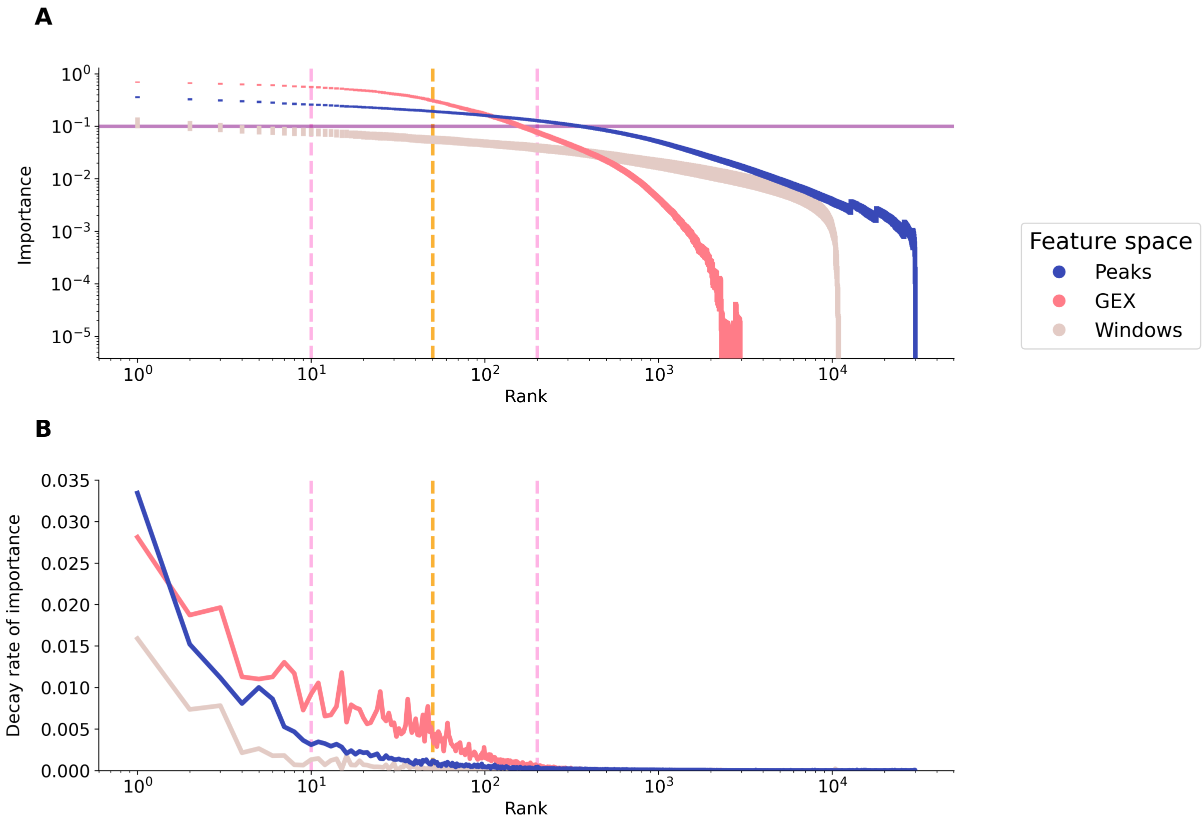

Fig. S7: **A** full range rankâĂŞimportance distribution of the features for each feature space. **B** full range derivative of the importance distribution of the features for each feature space.

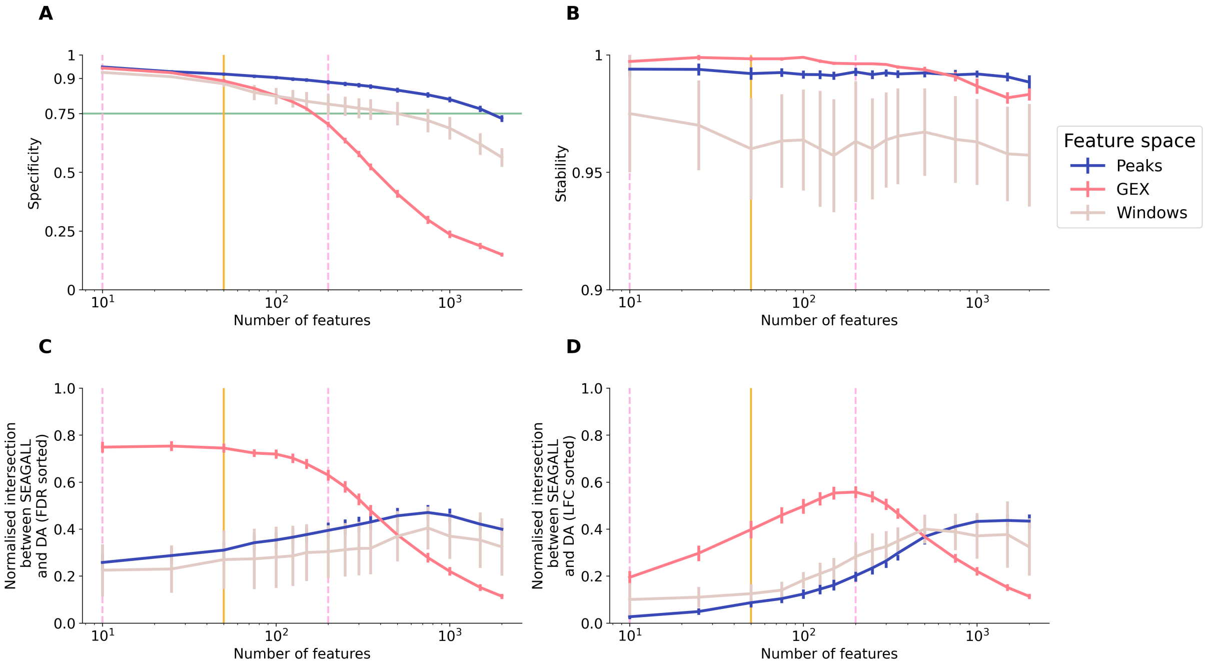

Fig. S8: **A** specificity of the explanations varying the number of features we asked the model to keep. Each colour represents one feature space; the mean value at each step is the average specificity for each cell type across all data sets after running the explainer 50 times. The error bar height represents the standard deviation of the mean. **B** stability of the explanation computed as described in A. **C**-**D** relative intersection between the XAIF and the DAF features varying the number of requested features.

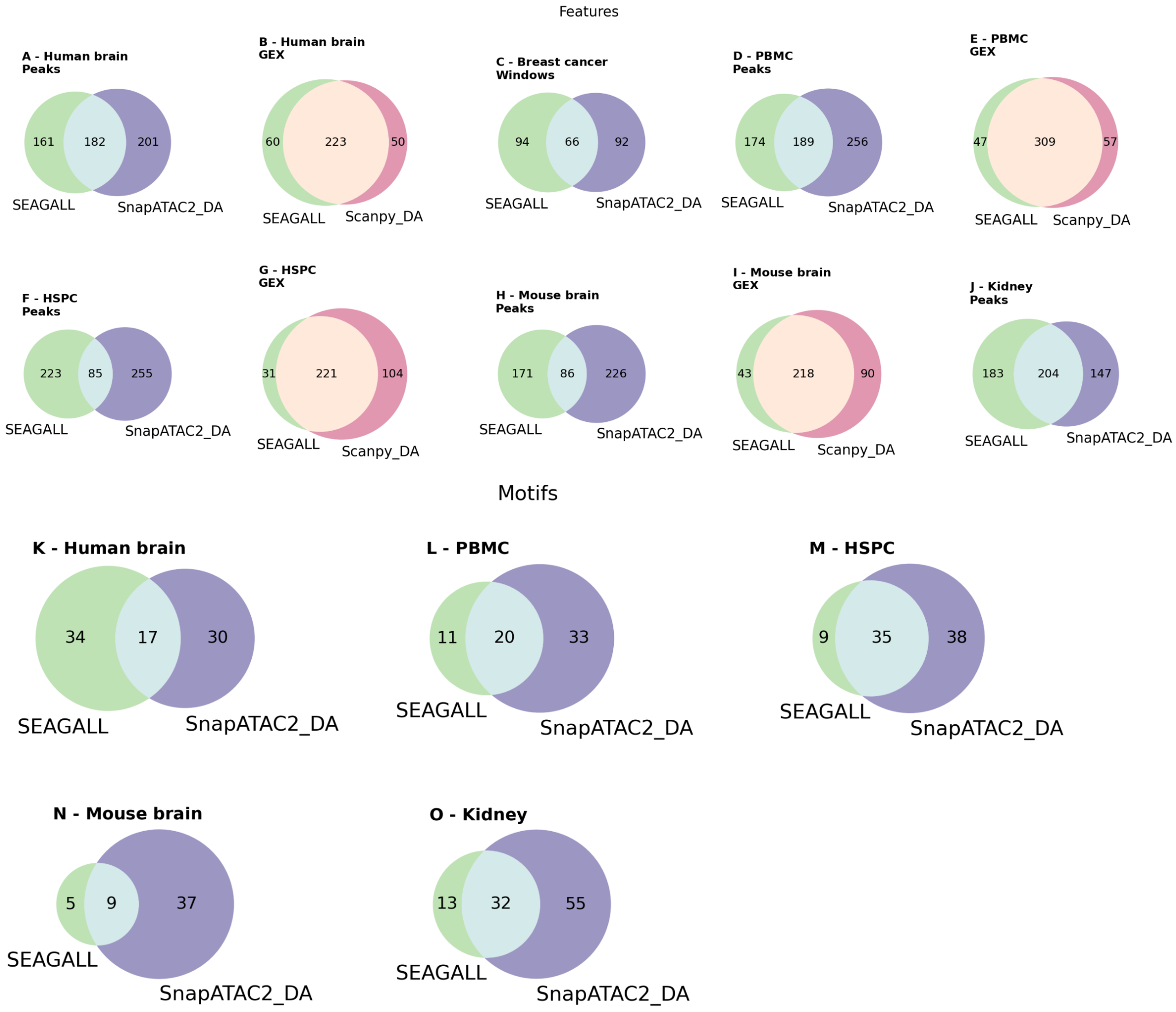

Fig. S9: **A-J** Venn diagrams quantifying the overlap between the SEAGALL features and those obtained with Scanpy or SnapATAC2 depending on the feature space. **K-O** Venn diagrams quantifying the overlap between motifs obtained from the features extracted with SEAGALL and those obtained by differential analysis with SnapATAC2.

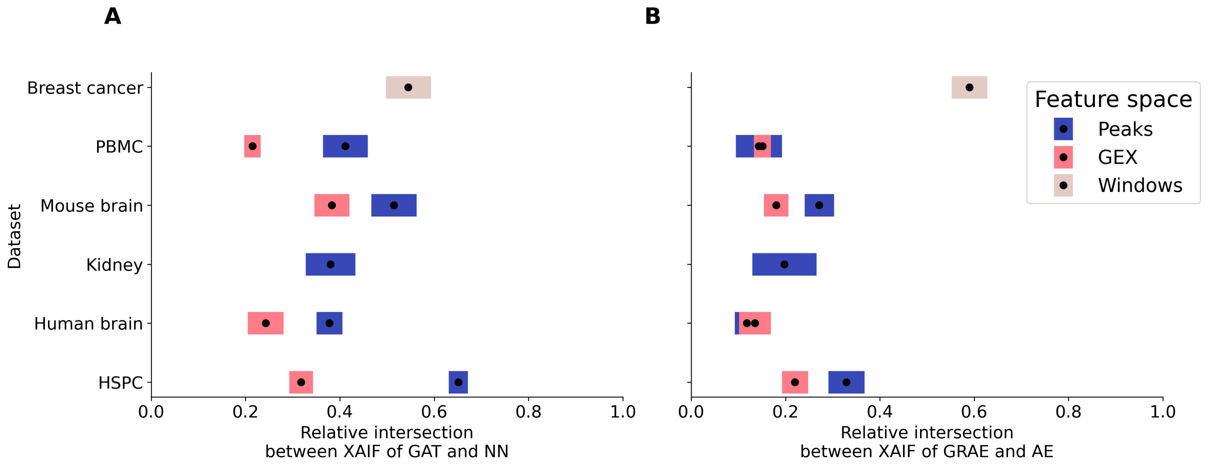

Fig. S10: **A** difference between the XAIFs of GAT and a normal NN without graph information. **B** difference between the XAIFs of GRAE versus AE for graph construction applying the same GAT. Removing either geometry or attention leads to different explanations.

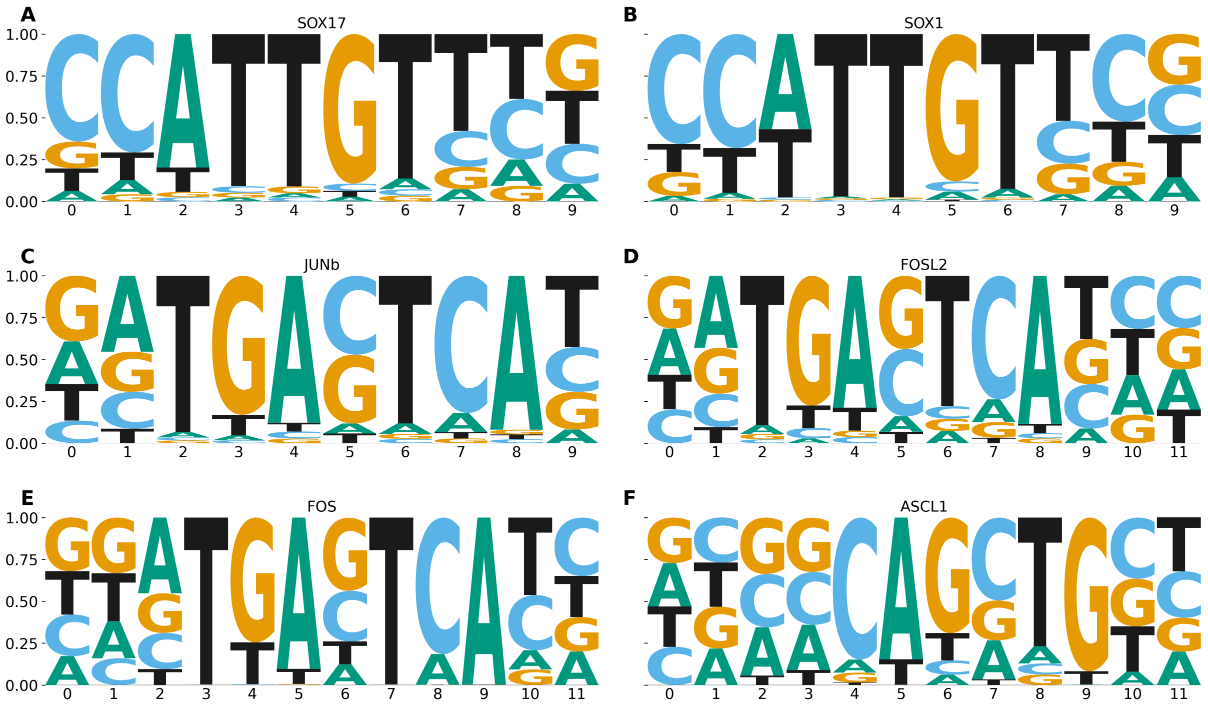

Fig. S11: **A**-**B** motif visualisation.

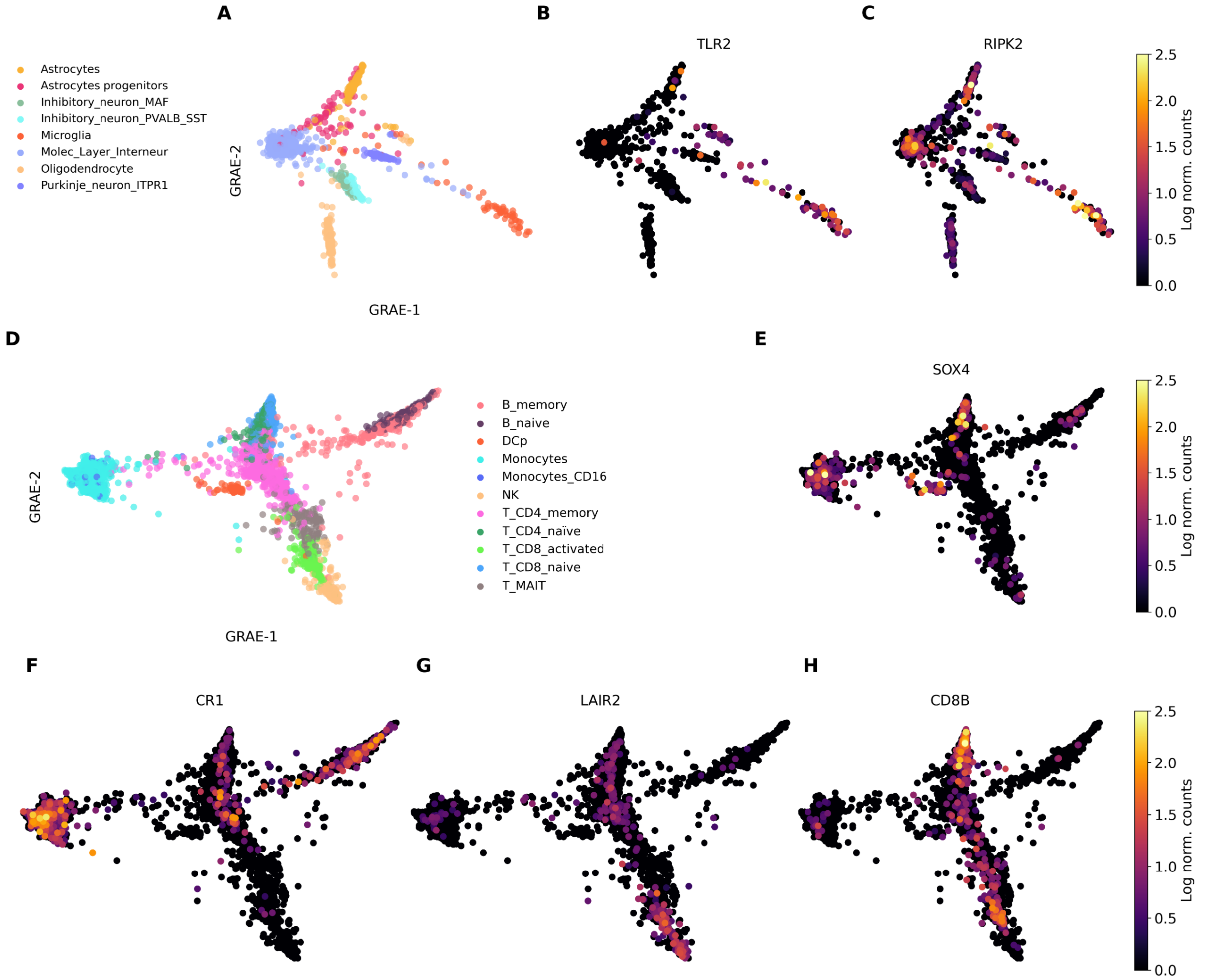

Fig. S12: **A** GRAE embedding of the scATAC human brain data set. **B**-**C** expression of TLR2 and RIPK2 in normalised log counts. **D** GRAE embedding of the PBMC GEX data set. **E**-**H** expression of SOX4, CR1, LAIR2 and CD8 (subunit B) in normalised log counts.

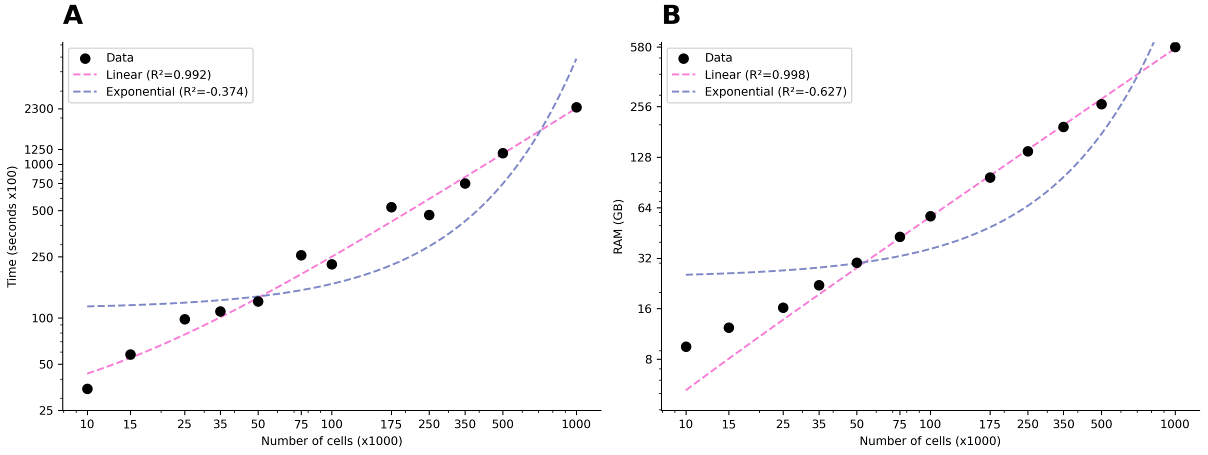

Fig. S13: **A** running time and **B** RAM usage of SEAGALL varying the number of cells. The algorithm scales linearly in both running time and RAM allocation, where N is the number of cells.

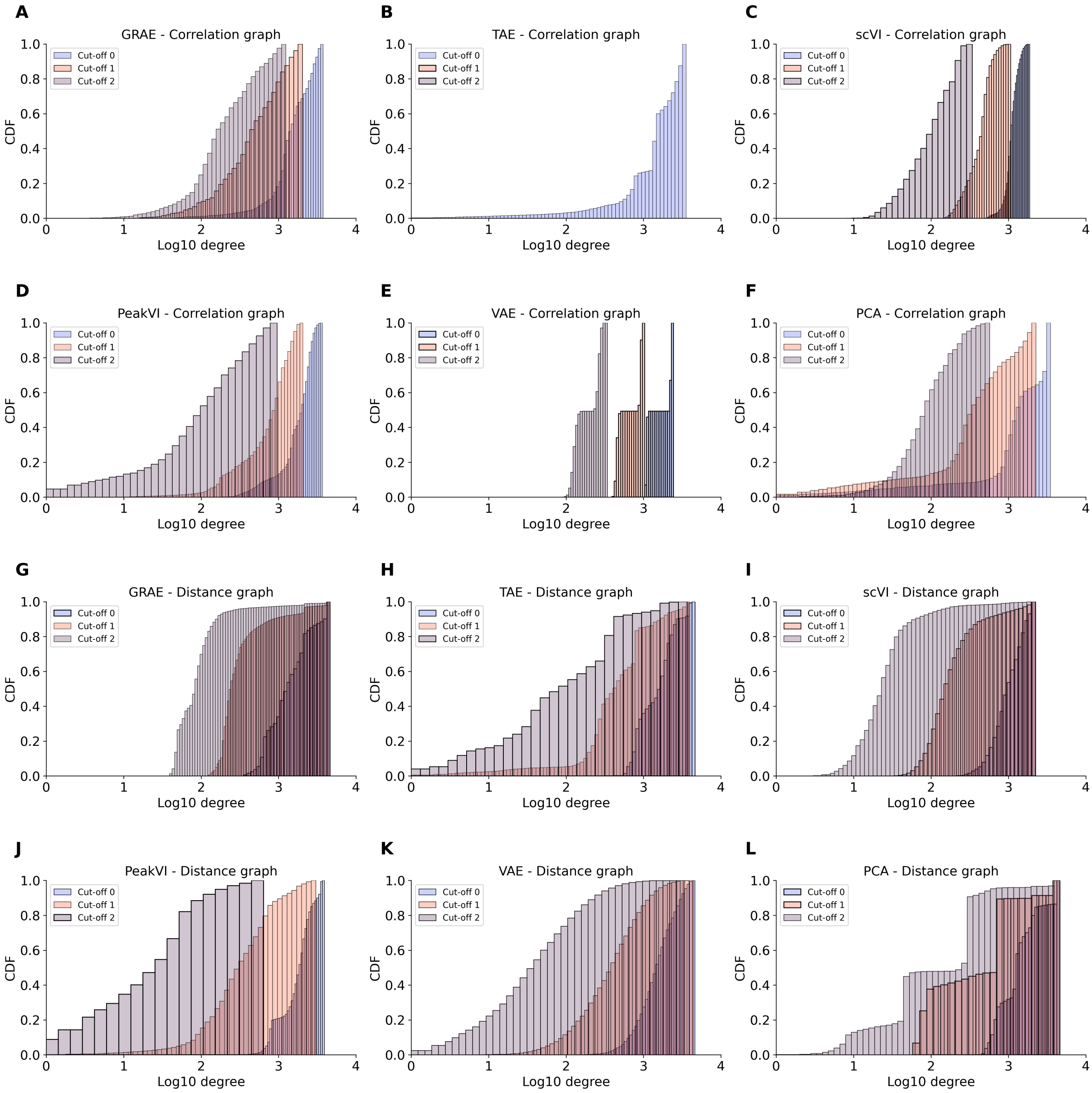

Fig. S14: **A**-**F** cumulative distribution function of the degree of the correlation graph varying the latent space and applying different cut-offs on the minimum correlation between nodes. **G**-**L** the same analysis based on Euclidean distance computed in the latent space.

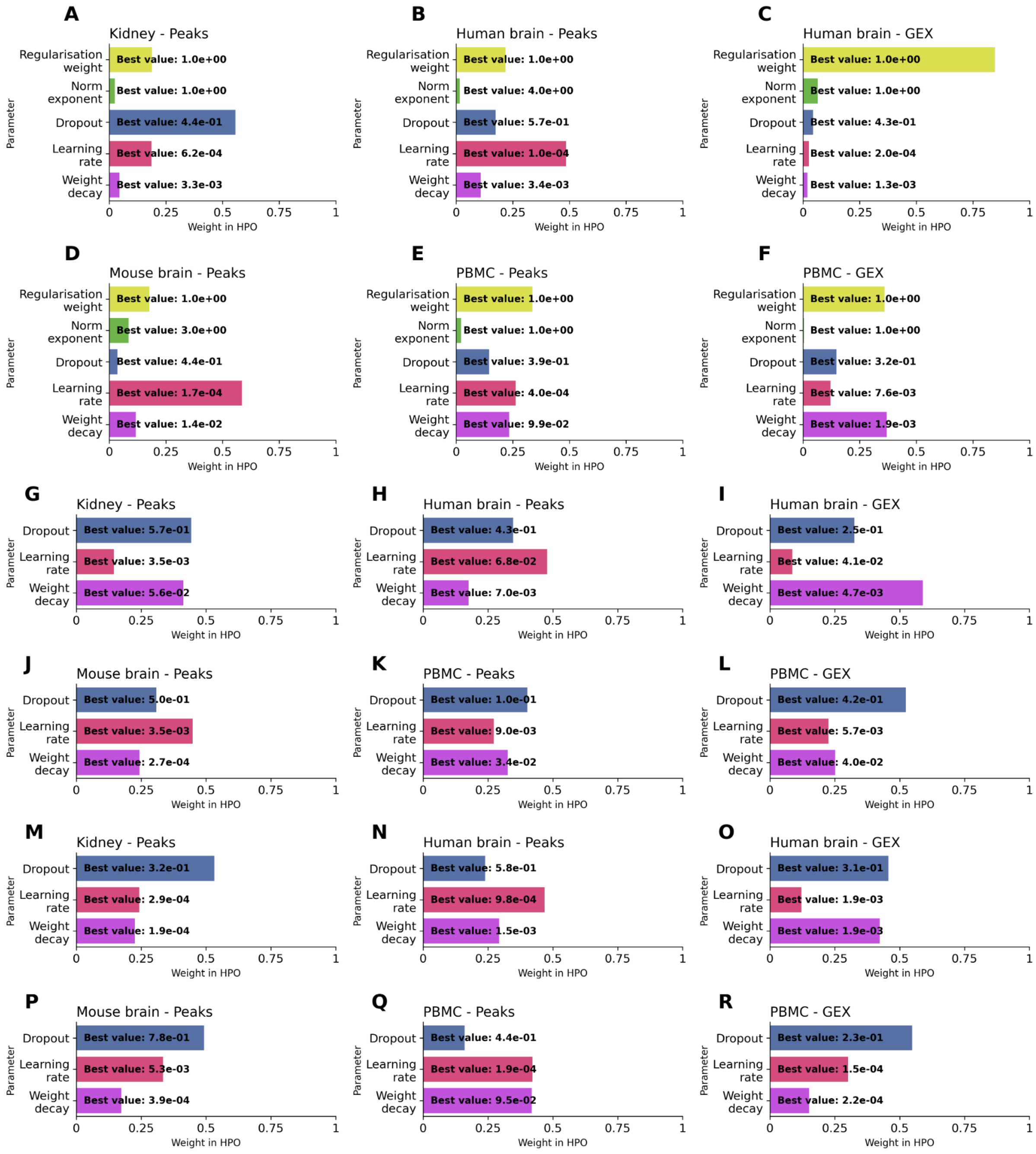

Fig. S15: Weight of each hyperparameter in the optimisation of TAE (**A**-**F**), VAE (**G**-**L**), AE (**M**-**R**). Each panel corresponds to one data set. The final value of each hyperparameter is the average across the six experiments.
