# Supplementary material for: Geometry-aware graph attention networks to explain single-cell chromatin states and gene expression with SEAGALL
Source: Genome Biol. 2026 Apr 23;27:188. doi: 10.1186/s13059-026-04066-2 (PMC13238118; doi:10.1186/s13059-026-04066-2)
Supplement: Supplementary file 4 — Additional file 4. Issues encountered during the attempted usage of competitor computational methods, particularly concerning their installation and operation. [file 13059_2026_4066_MOESM4_ESM.pdf]

Detailed report about the installation and running of other competing tools.

All the notebooks to reproduce these results can be found in Zenodo at doi:10.5281/zenodo.18788157

PAUSE

PAUSE: principled feature attribution for unsupervised gene expression analysis
Genome Biology 2023
https://genomebiology.biomedcentral.com/articles/10.1186/s13059-023-02901-4

The installation completes successfully. However, when proceeding to the second step of the tutorial, we encountered an issue. The tutorial instructs the user to call a function named `train`, passing two objects - `train_dataset` and `val_dataset` - that are not defined earlier in the workflow, and provides no guidance on how to construct them.

Based on a careful reading of the code, it appears that these objects are intended to be PyTorch `Dataset` instances, possibly with minor modifications, as suggested in the notebook *PAUSE_Test.ipynb* and in the `models.py` module. After manually creating appropriate dataset objects outside of the standard tutorial, we were able to proceed to the training step.

Unfortunately, execution stops shortly thereafter. Specifically, at line 907 of `models.py` (`loss = self.calc_loss(...)`), memory usage increases dramatically. Even with the dataset restricted to only two cells, more than 32 GB of RAM is allocated, causing the kernel to terminate.

To further investigate, we attempted to run the code on the LMU High Performance Computing cluster. In that setting, a single batch of 256 cells with 2,237 features required approximately 252 GB of RAM. To confirm that this behaviour occurs within a single epoch and a single batch, we added logging statements to report the epoch, batch, and training step (see the referenced notebook).

Despite these efforts, memory consumption remains extremely high, even when the batch size is reduced to 16 cells. An illustrative screenshot is attached for reference.

For reference, the full procedure and generated outputs are documented in the notebook *PAUSE_Test.ipynb*.

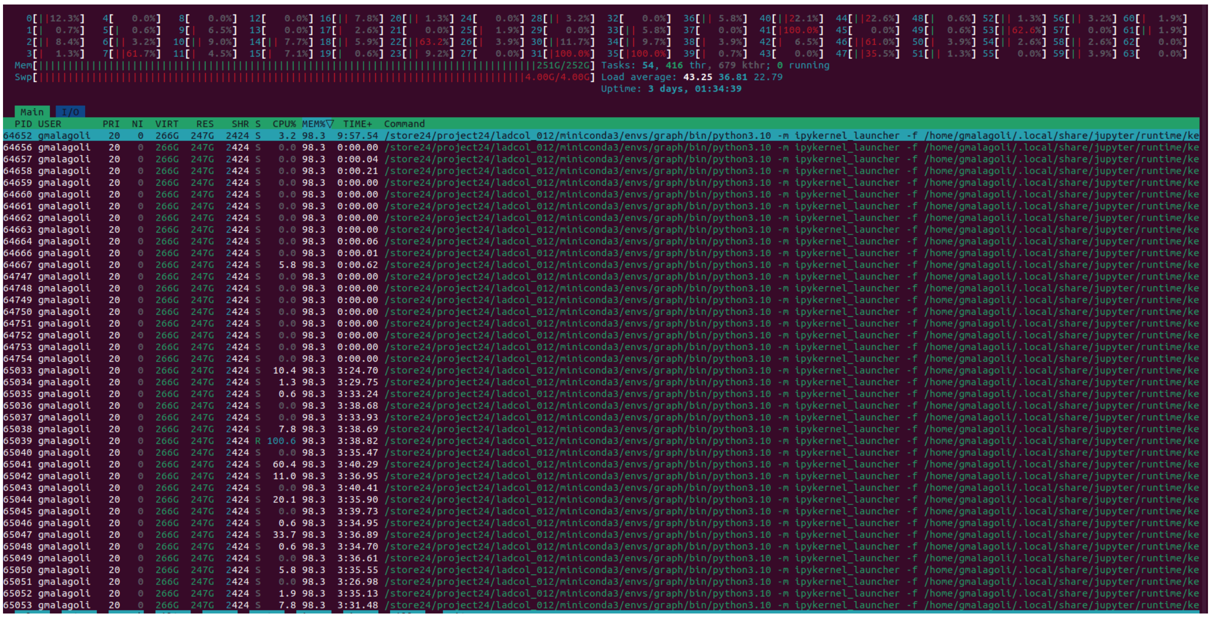

d-scIGM

Comprehensive single-cell RNA-seq analysis using deep interpretable generative modeling guided by biological hierarchy knowledge

Briefings in Bioinformatics 2024.

https://academic.oup.com/bib/article/25/4/bbae314/7705530

The installation completes successfully and the code executes without runtime errors. However, we encountered difficulties in interpreting both the input parameters and the resulting outputs.

Several command-line parameters - including `--rate`, `--topic_size`, `--hidden_size`, and `--embed_size` - share the same documentation string ("Number of units in hidden layer 1."). Because these parameters likely serve different purposes, identical descriptions make it difficult to understand their specific roles and adjust them appropriately.

We attempted to run the tool using the default parameter settings, modifying only the required input files. The structure of these inputs had to be inferred from the provided examples, as no detailed description of their expected format was available. Although the execution completes and produces output files, these outputs are not described in the tutorial or documentation, which makes it difficult to interpret or use the results.

For reference, the full procedure and generated outputs are documented in the notebook *d-scIGM_Test.ipynb*.

scASDC

scASDC: Attention Enhanced Structural Deep Clustering for Single-cell RNA-seq Data

IEEE International Conference on Bioinformatics and Biomedicine (BIBM)

https://ieeexplore.ieee.org/document/10822787

The installation completes successfully. However, execution stops immediately at the import stage due to missing functions (see notebook *scASDC_Test.ipynb*).

The initial import error can be resolved by appending the appropriate Python path to include the `utils` module. After addressing this, the code proceeds but then attempts to import two additional modules that are neither present in the GitHub repository nor available as installable packages (see attached image). As a result, it is unclear where these dependencies are expected to originate, and we are unable to proceed further.

We also noted that a related issue has been reported on the project's GitHub repository and remains unresolved at the time of writing (https://github.com/wenwenmin/scASDC/issues) [26/01/2026].

For reference, the full procedure and generated outputs are documented in the notebook scASDC_*Test.ipynb*.

```
[1]: import os

[2]: os.system("python run_scASDC.py")

Traceback (most recent call last):
  File "/store24/project24/ladcol_012/GNN/OtherMehtods/scASDC-master/run_scASDC.py", line 11, in <module>
    from utils import load_data, load_graph
ImportError: cannot import name 'load_data' from 'utils' (unknown location)

[2]: 256

[ ]:
```

1. Debugging

```
[3]: from __future__ import print_function, division
     import argparse
     import numpy as np
     from sklearn.cluster import KMeans
     import torch
     import torch.nn as nn
     import torch.nn.functional as F
     from torch.nn.parameter import Parameter
     from torch.optim import Adam
     from torch.nn import Linear

[4]: #Before it was: from utils import load_data, load_graph (but it did not work)
     from utils.utils import load_data, load_graph

[5]: from GNN import GNNLayer
     #GNNLayer do no existe anywhre in the GitHub nor is a required packaged according to requirements.txt

---------------------------------------------------------------------------
ModuleNotFoundError                       Traceback (most recent call last)
Cell In[5], line 1
----> 1 from GNN import GNNLayer
      2 #GNNLayer do no existe anywhre in the GitHub nor is a required packaged according to requirements.txt

ModuleNotFoundError: No module named 'GNN'

[6]: from utils.evaluation import eva
     #utils_ProtoMGAE do no existe anywhre in the GitHub nor is a required packaged according to requirements.txt

---------------------------------------------------------------------------
ModuleNotFoundError                       Traceback (most recent call last)
Cell In[6], line 1
----> 1 from utils.evaluation import eva
      2 #utils_ProtoMGAE do no existe anywhre in the GitHub nor is a required packaged according to requirements.txt

File /store24/project24/ladcol_012/GNN/OtherMehtods/scASDC-master/utils/evaluation.py:11
      9 import torch
     10 import torch.nn as nn
---> 11 from utils_ProtoMGAE import create_optimizer, accuracy
     12 from sklearn.cluster import KMeans
     14 torch.set_num_threads(1)

ModuleNotFoundError: No module named 'utils_ProtoMGAE'

[7]: from utils.preprocess import prepro, normalize_1

---------------------------------------------------------------------------
ImportError                               Traceback (most recent call last)
Cell In[7], line 1
----> 1 from utils.preprocess import prepro, normalize_1

File /store24/project24/ladcol_012/GNN/OtherMehtods/scASDC-master/utils/preprocess.py:14
     12 from sklearn.model_selection import train_test_split
     13 from . import utils
---> 14 from utils import decode, dotdict
     15 from sklearn.preprocessing import scale
     18 class AnnSequence:

ImportError: cannot import name 'decode' from 'utils' (unknown location)
```

scKAN

scKAN: interpretable single-cell analysis for cell-type-specific gene discovery and drug repurposing via Kolmogorov-Arnold networks

Genome biology 2025

https://genomebiology.biomedcentral.com/articles/10.1186/s13059-025-03779-0

The installation completed successfully, and we were able to run the preprocessing step on the dataset recommended in the tutorial without issues.

However, execution fails at the `stage1.py` step due to an import error indicating that a required module cannot be found (see notebook *scKAN_Test.ipynb*). The missing module, named `datasets`, does not appear as a file in the GitHub repository, nor is it available as an installable package. As a consequence, it is not possible to determine its intended location or to resolve the import manually.

We have opened a GitHub issue to request clarification (https://github.com/hehh77/scKAN/issues/1). At the time of writing (26/01/2026), the issue remains unresolved.

For reference, the full procedure and generated outputs are documented in the notebook scKAN_*Test.ipynb*.

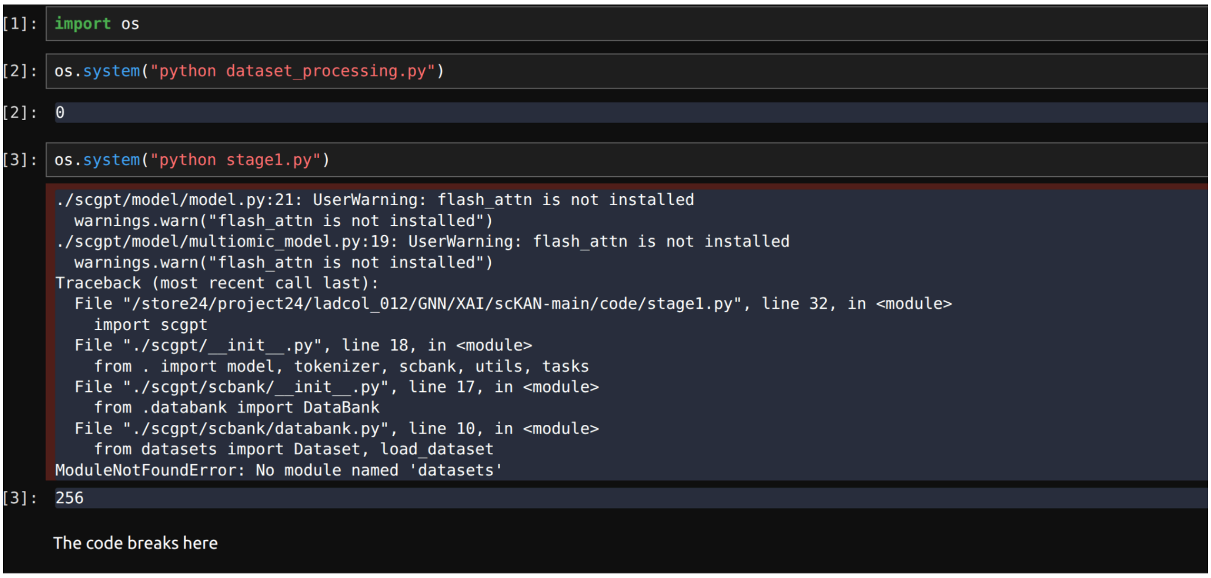

```
[1]: import os

[2]: os.system("python dataset_processing.py")

[2]: 0

[3]: os.system("python stage1.py")

./scgpt/model/model.py:21: UserWarning: flash_attn is not installed
  warnings.warn("flash_attn is not installed")
./scgpt/model/multiomic_model.py:19: UserWarning: flash_attn is not installed
  warnings.warn("flash_attn is not installed")
Traceback (most recent call last):
  File "/store24/project24/ladcol_012/GNN/XAI/scKAN-main/code/stage1.py", line 32, in <module>
    import scgpt
  File "./scgpt/__init__.py", line 18, in <module>
    from . import model, tokenizer, scbank, utils, tasks
  File "./scgpt/scbank/__init__.py", line 17, in <module>
    from .databank import DataBank
  File "./scgpt/scbank/databank.py", line 10, in <module>
    from datasets import Dataset, load_dataset
ModuleNotFoundError: No module named 'datasets'
[3]: 256

The code breaks here
```

scLEGA

scLEGA: an attention-based deep clustering method with a tendency for low expression of genes on single-cell RNA-seq data
Briefing in bioinformatics, 2024
https://academic.oup.com/bib/article/25/5/bbae371/7721463

The installation completes successfully. However, when running the provided test example, the code fails to open the input file used to evaluate the tool (see notebook *scLEGA_Test.ipynb*). As a result, we were unable to proceed with testing the workflow

For reference, the full procedure and generated outputs are documented in the notebook scLEGA_*Test.ipynb*.

```
[1]: import os
     os.system("python run_scLEGA.py")

     Traceback (most recent call last):
       File "/store24/project24/ladcol_012/GNN/OtherMehtods/scLEGA-main-master/run_scLEGA.py", line 85, in <module>
         adata, rawData, dataset, adj, r_adj = utils.load_data('./Data/AnnData/{}'.format(args.name), args=args)
       File "/store24/project24/ladcol_012/GNN/OtherMehtods/scLEGA-main-master/utils.py", line 43, in load_data
         adata = ad.read(dataPath + '.h5ad')
       File "/store24/project24/ladcol_012/miniconda3/envs/sckan/lib/python3.9/site-packages/anndata/__init__.py", line 60, in read
         return read_h5ad(*args, **kwargs)
       File "/store24/project24/ladcol_012/miniconda3/envs/sckan/lib/python3.9/site-packages/anndata/_io/h5ad.py", line 234, in read_h5ad
         with h5py.File(filename, "r") as f:
       File "/store24/project24/ladcol_012/miniconda3/envs/sckan/lib/python3.9/site-packages/h5py/_hl/files.py", line 564, in __init__
         fid = make_fid(name, mode, userblock_size, fapl, fcpl, swmr=swmr)
       File "/store24/project24/ladcol_012/miniconda3/envs/sckan/lib/python3.9/site-packages/h5py/_hl/files.py", line 238, in make_fid
         fid = h5f.open(name, flags, fapl=fapl)
       File "h5py/_objects.pyx", line 56, in h5py._objects.with_phil.wrapper
       File "h5py/_objects.pyx", line 57, in h5py._objects.with_phil.wrapper
       File "h5py/h5f.pyx", line 102, in h5py.h5f.open
     OSError: Unable to synchronously open file (file signature not found)

[1]: 256

[ ]:
```
